# Irregular Antibody Screening Using a Microdroplet Platform

**DOI:** 10.3390/bios13090869

**Published:** 2023-09-04

**Authors:** Ding-Ping Chen, Pei-Yu Wu, Yen-Heng Lin

**Affiliations:** 1 Department of Laboratory Medicine, Chang Gung Memorial Hospital, Taoyuan City 33305, Taiwan; 2 Department of Medical Biotechnology and Laboratory Science, College of Medicine, Chang Gung University, Taoyuan City 33302, Taiwan; 3 Department of Electronic Engineering, Chang Gung University, Taoyuan City 33302, Taiwan; 4 Department of Biomedical Engineering, Chang Gung University, Taoyuan City 33302, Taiwan

**Keywords:** droplet, microfluidics, irregular antibodies, manual polybrene test

## Abstract

The screening procedure for antibodies is considered the most tedious among the three pretransfusion operations, i.e., ABO and Rhesus (Rh) typing, irregular antibody screening/identification, and crossmatching tests. The commonly used screening method for irregular antibodies in clinics at present is a manual polybrene test (MP). The MP test involves numerous reagent replacement and centrifuge procedures, and the sample volume is expected to be relatively less. Herein, screening red blood cells (RBCs) and serum irregular antibodies are encapsulated in microdroplets with a diameter of ~300 μm for a hemagglutination reaction. Owing to the advantage of spatial limitation in microdroplets, screening RBCs and irregular antibodies can be directly agglutinated, thereby eliminating the need for centrifugation and the addition of reagents to promote agglutination, as required by the MP method. Furthermore, the results for a large number of repeated tests can be concurrently obtained, further simplifying the steps of irregular antibody screening and increasing accuracy. Eight irregular antibodies are screened using the proposed platform, and the results are consistent with the MP method. Moreover, the volume of blood samples and antibodies can be reduced to 10 μL and 5 μL, respectively, which is ten times less than that using the MP method.

## 1. Introduction

Blood is an important physiological fluid that regulates body temperature and exhibits immune functions. Red blood cells (RBCs) can be classified into 35 blood types according to their surface antigens [1,2]. The number of RBC surface antigens is a factor that affects immunogenicity. Generally, ABO antigens are the most common type of RBC antigen, followed by the Rh system. Therefore, the discovery of the ABO blood group system has greatly improved the safety of blood transfusions [3,4,5]. However, irregular antibodies may be responsible for causing transfusion reactions. Irregular antibodies refer to all antibodies except for the ABO blood group system that may form owing to the immune effects of blood transfusions or pregnancy in women [6,7,8,9]. Because irregular antibodies react with the corresponding antigens in the recipient, these antibodies induce the destruction of RBCs, causing chills, fever, or even hemolysis. Therefore, irregular antibody screening is an important step before blood transfusion.

Presently, an indirect anti-human globin test (IAT) is the primary method for screening irregular antibodies in clinical settings. This method was proposed by Coombs in 1945 [10] and entails using the subject’s serum to react with the screening RBCs containing known antigens. As most irregular antibodies undergo a weak agglutination reaction with screening RBCs, adding anti-human globulin to enhance the agglutination reaction is necessary. In cases when irregular antibodies are detected in the subject’s serum, the antibodies will adhere to the screening RBCs and agglutinate them via the immune reaction between anti-human globin and irregular antibodies. Detecting irregular antibodies in serum based on the IAT principle is currently realized through three platforms, namely, column antigen technology (CAT), solid-phase red cell adhesion assay (SPRCA), and manual polybrene test (MP) [11]. The CAT method was proposed by Lapierre et al. to test the binding of RBC blood cell antigens and antibodies [12]. In this method, the serum sample and antibody are mixed in a test tube, following which anti-human globulin is added to initiate the reaction. Following reaction completion, the sample is introduced into a column to separate the agglutinated and non-agglutinated RBCs [13]. According to the principle of column screening RBCs, it can be subdivided into two methods: using the pore size of the column gel to separate the RBC samples and modifying the column gel with proteins A and G having affinity for IgG antibodies; this is performed to ensure that the gel can adsorb the RBCs containing anti-human globulin [14,15]. The results of the two methods are nearly identical. In cases when an agglutination reaction occurs, the RBCs remain in the upper layer of the microcolumn, whereas the non-agglutinated RBCs remain at the bottom. The advantages of the CAT method are that the anti-human globulin test does not require washing and that the valence of agglutination can be quantified. However, the disadvantage is that a centrifuge is required to obtain the results following the reaction.

The method of assessing irregular antibodies using the SPRCA platform includes immobilizing known RBC antigens or RBCs on a microporous plate and adding the serum to be examined. If the relevant antibody is present in the serum, the antibody will bind to the antigen on the microplate. After adding the indicator red cells, if there are irregular antibodies in the serum, the indicator red cells will be evenly spread on the surface of the microplate following centrifugation; however, if no irregular antibodies are observed, the indicator red cells will be precipitated in the middle of the microplate following centrifugation [16]. The advantage of using SPRCA to detect irregular antibodies is that the detection can be completed without a test tube, which has the potential to be automated. The sensitivity and specificity are better than test tube methods. The disadvantage is the same as that of the CAT method, i.e., requiring a centrifuge to complete the detection.

The MP method can detect irregular antibodies in traditional test tubes, which was proposed by Lalezari et al. in 1980 [17]. Positively charged polymer molecular reagents, such as polybrene, are used to induce the nonimmune agglutination of RBCs to accelerate detection and improve sensitivity. The three to four types of screening RBCs with known antigens are used to react with irregular antibodies in serum. Adding positively charged polymer molecules can force all RBCs to approach first. The agglutination reaction between antigen and antibodies concomitantly occurs in an opportunistic manner, following which sodium citrate is used to neutralize the effect of polybrene. In cases where irregular antibodies are present, the agglutination will not spread. Since 1984, Taiwan has vigorously promoted the MP method to screen irregular antibodies in serum. The advantage is that the required equipment is simple and consistent with equipment commonly used in a laboratory. The disadvantages include that the operation procedures are cumbersome and the agglutination is not easy to quantify. Herein, microfluidic technology was used to simplify the MP method.

Microfluidic technology has been widely used in the fields of biochemical analysis and disease detection in recent years [18,19,20]. Owing to its small size, fast reaction, and automatic control, it can accelerate reaction completion and reduce sample volume. There have been numerous studies regarding ABO blood typing using microfluidic chips [21,22,23,24]. However, few studies focused on irregular blood typing. Chen et al. proposed a disk-like microfluidic chip for irregular antibody screening [25]. Furthermore, Chang et al. proposed another microfluidic chip for irregular antibody screening with a nanowire sensor [26,27]. Both of them performed all the MP test procedures for the typing. In addition, the microdroplet platform has been widely used [28,29,30]. Microreactors with a volume of tens of picolitres or nanoliters are formed using microchannels with two immiscible liquids. These microdroplets have the advantages of limiting the diffusion path, accelerating mixing, and simultaneous reactions. For example, DNA encapsulated in droplets can be used for a digital polymerase chain reaction, and the sensitivity of sensing can be improved by dispersing DNA samples into droplets [31,32]. In addition, cells can be encapsulated into microdroplets to perform single-cell metabolic studies, including those of lactate and pyruvate released by cancer cells [33,34], cell secretion of antibodies, and cell–cell interactions [35]. Using microdroplets can confine cells into a small space, thereby accelerating the reaction response.

In addition to the abovementioned application of microdroplets encapsulating DNA or cells, blood agglutination assays have been performed in microdroplet environments. To illustrate this, Marcali and Elbuken proposed impedimetric sensing of a droplet system that can detect blood agglutination in droplets [36]. Furthermore, a digital microfluidic device with droplet manipulation was used for blood agglutination assays [37]. Moreover, the current study group utilized a similar concept to study the mechanism of mixed-field agglutination in subtypes [38]. These studies showed the potential of the microdroplet platform for blood typing. However, these studies only demonstrated regular ABO and Rh typing or subtyping. The proposed method demonstrates the potential of irregular blood typing in the microdroplet platform.

The agglutination reaction between irregular antibodies and corresponding RBCs is relatively weak, and the detection of irregular antibodies via the MP method is cumbersome. Therefore, herein, we used the merits of the microdroplet platform to increase the probability of collision between RBCs and irregular antibodies within a limited volume, thus simplifying the MP method procedures. In addition, this method can generate hundreds of microreactors that do not interfere with each other at the same time, thereby ensuring the high reproducibility of the experiment results. Moreover, it can reduce the use of blood samples and reagents.

## 2. Materials and Methods

### 2.1. Chip Design and Fabrication

**Figure 1:**
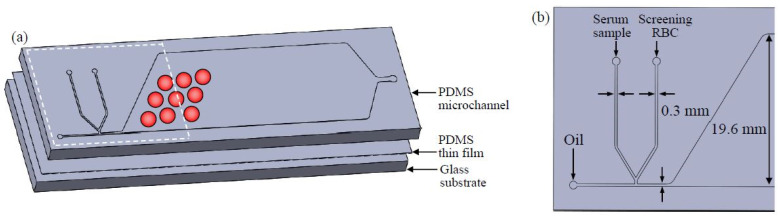
(**a**) Design of the microdroplet platform, where the PDMS with a microchannel is the upper layer and a glass slide with a thin PDMS is the lower layer. (**b**) Detailed design of the microdroplet generation zone, with a flow channel width of 0.3 mm, observation zone width of 19.6 mm, and flow channel height of 0.1 mm. Serum and screening RBCs are converged from the two flow channels and subsequently cut off by fluorinated oil to form microdroplets, allowing the samples to react within the microdroplets.

A T-shaped microchannel was used to generate microdroplets where the agglutination reaction of RBCs occurred. The irregular antibodies and serum samples were used as the aqueous phases, and fluorinated oil was used as the oil phase. The microfluidic chip comprised three layers. The top layer comprised poly-dimethylsiloxane (PDMS; SYLGARD 184, Dow Corning, Midland, MI, USA) with a microchannel and an observation area, and the bottom layer was a glass substrate with a PDMS film, which was used to ensure that the surface properties remained consistent throughout the flow channel (Figure 1a). A master mold was used to create the PDMS structure in the upper layer. The master pattern structure was prepared by coating negative photoresist SU-8 (2050, MicroChem, Adel, GA, USA) at 500 rpm for 10 s and 1800 rpm for 30 s (MS-A100, MIKASA, Tokyo, Japan) on a 4” wafer and fabricating a 100 µm high structure through a standard photolithography process. After the master pattern was completed, the PDMS A and B agents were mixed in a ratio of 10:1 and poured into the master mold, following which the mold was placed in an oven (DO60, DENG YNG, New Taipei City, Taiwan) at 80 °C for ~30 min to cure the PDMS. The cured PDMS was demolded and holes were punched at the inlet and outlet locations (Biopsy punch, ProSciTech, Kirwan, QLD, Australia). The lower layer was created by pouring PDMS onto a 25 × 75 mm glass substrate, coating at 1000 rpm for 10 s, baking at 80 °C for ~30 min, and bonding the upper and lower layers of PDMS with oxygen plasma (PDC-001, Harrick Plasma, Ithaca, NY, USA). To allow the formation of water-in-oil droplets in the microchannel, the PDMS flow channel surface was modified to be hydrophobic using Aquapel (PPG Industries, Pittsburgh, PA, USA) [39]. The chip size was 75 × 25 mm. Figure 1b shows the design of the microchannel. As the irregular antibody screening must mix serum and RBCs, the design of the dispersed phase flow channel was a Y-shaped side channel, with the serum sample flowing through from one side of the channel and the RBCs flowing through from the other side of the channel where they converged into the main flow channel in the continuous phase. The microdroplets were formed with the help of a shear force generated by the oil phase. The width of both the side channel and the main channel was 0.3 mm, which can produce hundreds of microreactors for RBC agglutination within a few seconds. The microdroplets flowing through the progressively wider flow channel (19.6 mm) can slow down the flow velocity to assist the agglutination observation and observation of more droplets per field of view.

### 2.2. Experimental Principle and Design

The irregular antibody screening method used in this study was based on the MP method proposed by Lalezari [17]. The principle of the MP method is to use three types of RBCs (SI, SII, and SIII) with known surface antigens to agglutinate with irregular antibodies in serum. These three types of screening RBCs each carry ~20 common RBC antigens. Following the reaction between the serum testing antibody and the screening RBCs, the types of irregular antigens can be initially screened by assessing the agglutination of the RBCs against the antibody-screening table provided by the screening reagent manufacturer. Three main steps are involved in performing the MP method in a test tube: (1) using a solution with a low ionic medium (LIM) to reduce the zeta potential of the RBCs, thus making it easier for the surface antigens to bind to the antibodies and bring the RBCs closer to each other; (2) adding polybrene and performing centrifugation to produce a nonspecific aggregation of RBCs; and (3) dispersing this nonspecific agglutination using trisodium citrate solution to observe the real agglutination caused by an antigen–antibody reaction. The purpose of these three steps is to accelerate the RBC agglutination reaction; however, for the operator, the entire test requires the addition of reagent thrice, and some of these reactions can only be observed following centrifugation. This study used the advantage of the microdroplet platform to confine the agglutination reaction to a limited space (tens of nanoliters), via which more than hundreds of reactions can be observed simultaneously. We assumed that in a limited space, the RBC agglutination reaction would not require the addition of LIM, polybrene, and trisodium citrate solutions or centrifugation to speed up the reaction and that encapsulating the serum sample and screening RBCs in water-in-oil microdroplets would suffice for the reaction. This would simplify the operation, limit the use of reagents, and eliminate the need for a centrifuge. First, the experiments were performed by varying the ratio of the flow rate of the continuous phase to produce droplets of different sizes. Next, three screening RBCs—SI, SII, and SIII—were reacted with eight irregular antibodies in microdroplets and observed for agglutination. Finally, the Anti-D antibody was serially diluted to test the reaction between different concentrations of irregular antibodies and screening-use RBCs and observe the agglutination time. Finally, we modified the chip design to allow the droplets to flow through two periodic flow channel heights, which deformed the droplets periodically to accelerate the agglomeration rate.

### 2.3. Experimental Setup and Reagent Preparation

**Figure 2:**
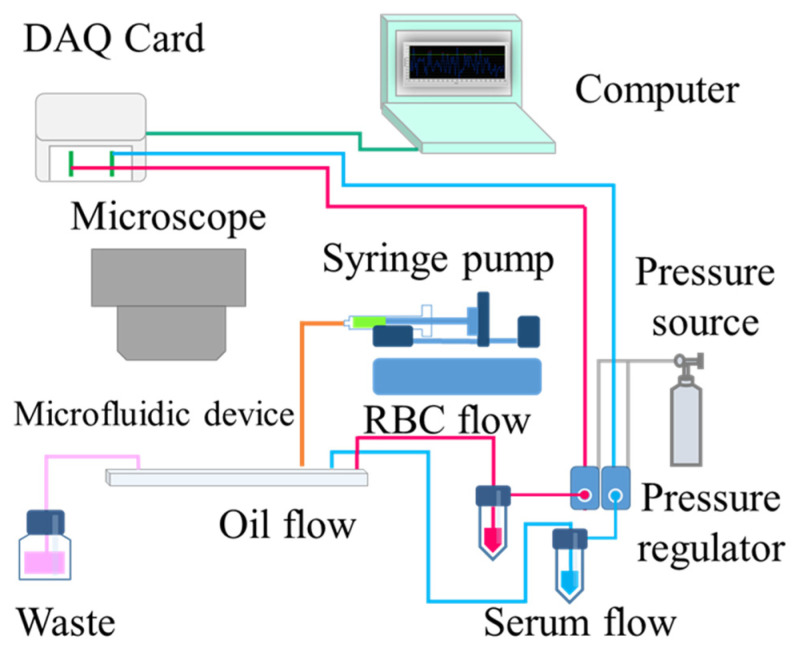
Schematic diagram of the experimental setup. The flow rate of RBCs and serum is controlled using pneumatically driven fluid, and the flow rate of fluorinated oil is controlled by the syringe pump, which can produce microdroplets of uniform size. The sample is allowed to react within hundreds of microdroplets, and the reaction in the microdroplets is recorded and observed using a microscope and digital camera.

The dispersed phase, serum sample, and screening RBCs were injected into the Y-shaped side channel using a pneumatically driven setup. The pressure of the regulator valve (VSO-EP-Miniature Pressure Controller, Parker, USA) was controlled using software (Labview, National Instruments, USA) through a DAQ card (Data Acquisition, USB-6281, National Instruments, USA), and the flow rate could be adjusted by fluctuating the air pressure. To avoid pressure interference between the dispersed phase and continuous phase, the fluorinated oil (with 2% home-synthesized inert block copolymer surfactant) was injected into the main flow channel using a syringe pump (Legato 180, KD Scientific Inc., Holliston, MA, USA). The dispersed phase was cut off to form microdroplets, and blood agglutination was observed using a microscope (BX 40, Olympus, Tokyo, Japan) mounted with a digital camera (ILCE-5100Y, Sony, Tokyo, Japan). We used 4× (UPLFLN4XPH, Olympus, Tokyo, Japan) and 20× (LCACHN20XPH, Olympus, Tokyo, Japan) objective lenses for image acquisition. A schematic diagram of the experimental setup is shown in Figure 2. The following reagents were used in the experiments: RBC MeDiPro Antibody Screening Cell (SI, SII, and SIII), which was purchased from Formosa Biomedical (Taipei, Taiwan); irregular antibodies Anti-D, Anti-C, Anti-E, Anti-ce, Anti-ec, Anti-Le^a^, Anti-S, and Anti-M, which were procured from Sanquin (Amsterdam, the Netherlands); fetal bovine serum (TMS-013-BKR), which was purchased from EMD Millipore (Burlington, VT, USA); and fluorinate oil HFE-7500, which was purchased from 3M (Saint Paul, MN, USA). In terms of the irregular antibody sample preparation, it is known that in the agglutination reaction, what is most important is the intact antigen on the RBC surface and not the number of antibodies. Thus, the spiked serum sample was obtained by mixing fetal bovine serum with commercially available antibodies in a volume ratio of 1:1.

## 3. Results and Discussion

### 3.1. Control of Sample Volume Ratio and Droplet Size

**Figure 3:**
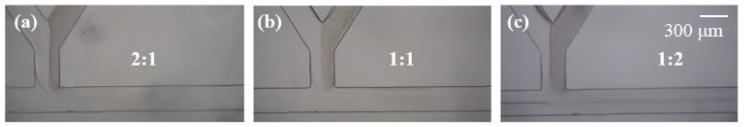
Volume ratio of serum to screening RBCs in the microdroplet is controlled using a Y-shaped flow channel and the sample flow rate. (**a**) The serum-to-RBC volume ratio of 2:1. (**b**) Serum-to-RBC volume ratio of 1:1. (**c**) Serum-to-RBC volume ratio of 1:2.

To achieve a 2:1 volume ratio of serum to screening RBCs, as in the MP method, the flow rate of the fluorinated oil in the continuous phase was initially fixed at 30 µL/min, and the flow rates of the serum sample and screening RBCs were adjusted individually by applying air pressure. The serum sample passed through the left side of the Y-shaped flow channel, and the screening RBCs passed through the right side of the flow channel. The experimental results are shown in Figure 3. The two samples were in a laminar flow in the Y-shaped microchannel; Figure 3a–c show the serum sample and the screening RBCs in the microchannel when the volume ratios were 2:1, 1:1, and 1:2 and the driving air pressure was 0.62:0.58, 0.62:0.62, and 0.58:0.62 psi, respectively. These ratios determined the ratio of the sample encapsulated in the droplet, and the Y-shaped flow channel design made it easy to change the volume ratio of the two samples in the droplet.

**Figure 4:**
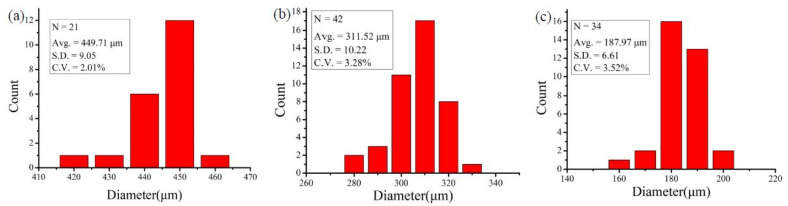
Microdroplet size control and uniformity analysis. Homogeneity analysis of microdroplets in the following three diameters: (**a**) 450, (**b**) 311, and (**c**) 188 µm.

Then, the driving air pressure of the dispersed phase was fixed, and the droplet size was changed by changing the flow rate of the fluorinated oil in the continuous phase. The average microdroplet sizes of 449.71, 311.52, and 187.97 µm were produced using fluorinated oil flow rates of 30, 60, and 80 µL/min, respectively, and the uniformity of microdroplet sizes was measured. The coefficient of variation in microdroplet size was within 3.52%, demonstrating that the microdroplet size can be regulated and a fairly uniform microdroplet size can be obtained (Figure 4a–c). In general, the reaction of serum and screening RBCs encapsulated in microdroplets has the following advantages over reaction in tubes: no LIM, centrifugation, and trisodium citrate required to accelerate the reaction, which is convenient for practical uses.

### 3.2. Eight Irregular Antibodies and Screening RBC Tests in Microdroplets

**Figure 5:**
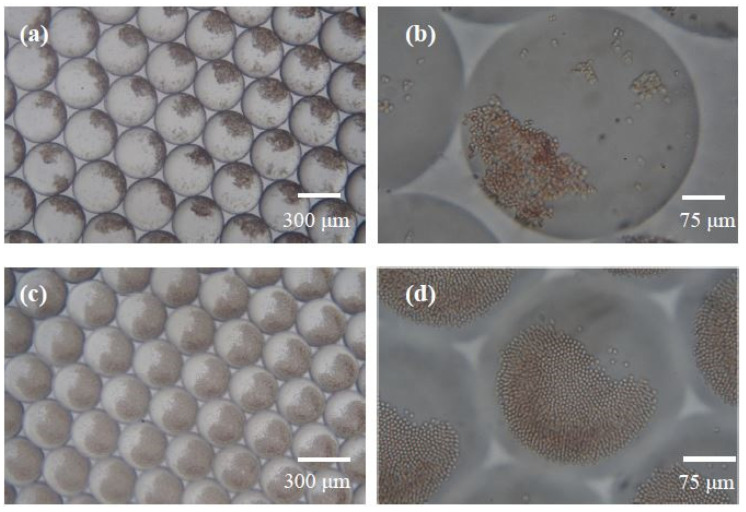
Irregular antibodies react with screening RBCs to form agglutinated and non-agglutinated images within the microdroplet. (**a**) Images of agglutination in microdroplets after 15 min of reaction between Anti-E and SI (4× objective). (**b**) Images of RBC agglutination under a 20× objective for the abovementioned reaction conditions. (**c**) Image of non-agglutination in microdroplets after 15 min of reaction between Anti-E and SII (4× objective). (**d**) Images of the abovementioned reaction conditions under a 20× objective.

First, we defined the observation criteria for the agglutination of RBCs in the microdroplets. The irregular antibody Anti-E and screening RBC SI were encapsulated in the microdroplets as the positive reaction sample, whereas the serum not containing any antibody was encapsulated in a droplet with screening RBCs SI as the negative reaction sample. Figure 5 shows the results of the reaction between Anti-E and RBC SI after 15 min, with microscopic objective magnifications of 4× and 20×. The agglutination reaction appeared to simultaneously occur in all the microdroplets through the 4× objective (Figure 5a). In the agglutinated RBCs, darker RBCs can be observed clustered in one corner of the microdroplet. To observe the agglutination of RBCs more clearly, a 20× objective was used. The agglutinated RBCs formed a coagulated structure, and the morphology of each RBC was no longer clear (Figure 5b). Figure 5c,d reveal the results of the reaction between the serum without irregular antibody and screening RBC SI after 15 min. The results observed using a 4× objective revealed that the non-agglutinated RBCs precipitated at the bottom of the microdroplet because of gravity (Figure 5c), whereas the results at a microscopic magnification of 20× revealed that the RBCs were close together owing to precipitation at the bottom of the microdroplet (Figure 5d); however, the appearance of each RBC can still be distinct from each other. One significant difference between the agglutinated RBCs and non-agglutinated RBCs in the microdroplet is the RBCs’ morphology observed through a microscopic magnification of 20×. Every RBC boundary can be observed when the agglutinated reaction does not take place. However, this feature is not easily quantitated through image analysis software. In this study, the images were manually distinguished. However, we aimed to develop an automatic image recognizer using machine learning technology. Deep Network Designer (The MathWorks, Inc., USA) was used to adapt a pretrained GoogLeNet network to classify the agglutination and non-agglutination of images. Preliminary data show that the more complete the agglutination process images for training are, the more accurate the recognition that can be achieved. By using machine learning real-time image recognition, human errors may be reduced.

Next, the eight irregular antibodies Anti-D, Anti-C, Anti-E, Anti-c, Anti-e, Anti-Le^a^, Anti-S, and Anti-M were prepared and tested for the agglutination reaction with the three screening RBCs SI, SII, and SIII using a microdroplet platform. Serum and RBCs were encapsulated in microdroplets with a diameter of ~300 μm in a volume ratio of 2:1. The reaction was recorded continuously under a microscope for 15 min, and the time required for the RBC agglutination to reach the steady state was analyzed by capturing the images from the video. The experimental results are shown in Table 1, where the reactions that could lead to RBC agglutination were labeled as Y with the time required to form steady-state agglutination, and those that could not were labeled as N. The agglutination results of the eight irregular antibodies with the three screening RBCs in the microdroplets are consistent with the antibody-screening table provided by the screening RBC manufacturer, thereby demonstrating the potential for the rapid and easy screening of irregular antibodies using this platform. The screening with the same samples was performed simultaneously using the MP method in tubes, and the results are also consistent with the reaction on the microdroplet platform. 

A comparison of the operation procedure, reagents and volume use, and consumption time between the proposed microdroplet platform and conventional MP test in a tube is depicted in Table 2. Using the microdroplet platform can not only omit the tedious operations and high reagent consumption, such as LIM, polybrene, and sodium citrate, of the MP method but also reduce the sample volume, thereby benefiting certain patients, such as newborn babies. The time required for both methods was broadly the same (within 10 min).

The experimental results revealed that the reaction time between Anti-D and the three types of screening RBCs was faster than that of other irregular antibodies, taking ~3 min to form steady-state agglutination, thus indicating that Anti-D exhibits a strong affinity for the D antigen on the surface of the three types of screening RBCs. The other irregular antibodies that can form agglutination with the screening RBCs all formed steady-state agglutination at ~6 min. Regarding combinations that failed to form agglutination, we did not observe after agglutination 15 min or even 1 h of reaction. The time series of the agglutination reaction of Anti-D, Anti-C, Anti-E, Anti-c, Anti-e, Anti-Le^a^, Anti-S, and Anti-M along with the three types of screening RBCs is presented in Supplemental Figures S1–S8. All images were acquired under 4× objective lens.

### 3.3. Agglutination Test for Diluted Anti-D Antibody and Microchannel Structure for Reaction Acceleration

Considering that the concentration of irregular antibodies in human blood is not easy to quantify, the spiked sample used in the above experiment was a mixture of fetal bovine serum and commercially available antibodies in a volume ratio of 1:1. To evaluate the detection limit of this microdroplet platform against irregular antibody concentration, we further diluted the above-configured Anti-D spike sample with fetal bovine serum to 256 times in a four-fold volume series and performed agglutination tests with screening RBCs SI, SII, and SIII on the microdroplet platform (Table 3). The time required for the four-fold diluted Anti-D antibody to react with screening RBCs SI, SII, and SIII individually to reach steady-state agglutination was 3 min (Supplemental Figure S9), which was similar to the time required for the original spike samples. When Anti-D was diluted 16 and 64 times (Supplemental Figures S10 and S11), the time required to reach steady-state agglutination was ~4 min. When Anti-D was further diluted to 128 and 256 times, the reaction times required to reach steady-state agglutination with the screening RBCs SI, SII, and SIII were 10 and 15 min, respectively (Supplemental Figures S12 and S13). Therefore, the lower the antibody concentration, the longer the time required for agglutination, and when the antibodies were diluted 256 times, the number of antibodies corresponding to the number of RBCs was still sufficient; however, the overall rate of the agglutination reaction was slowed down by the decrease in antibody concentration.

In addition, to further shorten the agglutination time, we accelerated the agglutination reaction by changing the design of the microchannel structure (Supplemental Figure S14a) so that the original flow channel design remained unchanged, and only the design of the top cover of the flow channel was altered from a flat surface to a structure with periodic grooves. The depth of the grooves was 100 µm, the width was 200 µm at the top and 400 µm at the bottom, and the spacing between each groove was 500 µm. As the height of the original flow channel was 100 µm and the microdroplet size was ~300 µm in diameter, the microdroplet was periodically released and squeezed when flowing through the groove structure (Supplemental Figure S14b), thereby causing a disturbance in the fluid inside the microdroplet and increasing the agglutination reaction rate. The reason for using the trapezoidal structure was that the microdroplets could pass through the structure smoothly without being trapped in the structure. We chose an Anti-M antibody that exhibited a weaker affiliation with the screening RBC surface antigen to evaluate the effect of the microstructure. Figure S15a–c show the reaction of Anti-M with SI, SII, and SIII following microdroplet formation in the accelerated structure, respectively. The experimental results demonstrated that the reaction time for the reaction of SI, SII, SIII, and Anti-M to reach steady-state agglutination was ~5 min 15 s to 5 min 20 s, which reduces the reaction time by ~16.7% compared with the flow channel without accelerated structure. The agglutination reaction can be further accelerated by using a flow channel structure that deforms the microdroplets.

## 4. Future Perspectives

The proposed platform has several advantages over the traditional MP method. However, some potential limitations need to be addressed in actual clinical settings, in terms of point-of-care use, in particular. In the present study, a pressure-based microfluidic controller was used to generate the microdroplets. The size of the controller was relatively bulky and not portable. Some methods of finger-powered droplet generation can be implemented for point-of-care use. Furthermore, we observed agglutination in droplets using bulky instruments. The commercially available mobile phone magnification lens attachment may be used to replace a bulky microscope. In addition, we believe that the scalability of the chip production and the reproducibility of the agglutination reaction could not be considered the main challenges to the implementation of the proposed method in clinical use. However, the accuracy and convenience of image recognition are key factors for practical application. Image recognition via manual methods is not convenient and relies too heavily on an experienced person in this study. We aim to develop an automatic image recognition method using machine learning technology.

## 5. Conclusions

Herein, a microdroplet platform was used for screening irregular antibodies in serum, and an RBC agglutination reaction was performed by encapsulating RBCs and serum in microdroplets of ~7–10 nL in size. The operation procedure has several advantages over the MP method in test tubes: (1) there is no need to use LIM reagents, (2) there is no need to perform centrifugation, (3) there is no need to use trisodium citrate reagents, (4) a large number of repetition tests can be performed in each microdroplet simultaneously, and (5) the technique can be multiplexed to screen several different antibodies within a single run if the chip is designed to have three individual droplet generation channel for the three screening RBCs. The results obtained for the agglutination reaction were consistent with the antibodies screening table provided by the manufacturer of the screening RBCs, and the detection time of irregular antibodies was slightly faster than that of the MP method. The study outcomes revealed that each irregular antibody exhibited a different antigen affinity toward the surface of the screening RBCs, resulting in some differences in the time required for RBC agglutination. In addition, for the same type of irregular antibody, the time required for agglutination is proportional to the concentration of the antibody. Finally, the design of the microchannel allows the microdroplets to accelerate the agglutination reaction by repeat deformation, which is estimated to reduce the overall reaction time by 16.7%. Thus, this method can be used to develop a simple, rapid, and low-cost screening platform for irregular antibodies.

## Figures and Tables

**Table 1:** Agglutination results of 8 irregular antibodies reacting with RBCs SI, SII, and SIII for screening.

	Ab	Anti-D (Figure S1)	Anti-C (Figure S2)	Anti-E (Figure S3)	Anti-c (Figure S4)	Anti-e (Figure S5)	Anti-Le^a^ (Figure S6)	Anti-S (Figure S7)	Anti-M (Figure S8)
RBC Agglutination									
SI		Y (3 min)	N	Y (6 min)	Y (6 min)	N	Y (6 min)	N	Y (6 min)
SII		Y (3 min)	Y (6 min)	N	N	Y (6 min)	N	Y (6 min)	Y (6 min)
SIII		Y (3 min)	Y (6 min)	N	N	Y (6 min)	N	N	Y (6 min)

**Table 2:** Comparison of the procedure, reagent used, volume and time between proposed microdroplet platform, and conventional MP test in a tube.

Microdroplet Platform				MP Test in Tube			
**Procedure**	Droplet Generation		Incubation	Sample Preparation	Promote Reaction	Form Nonspecific Binding	Resuspend RBC
**Reagent or sample**	Serum + RBC	Oil	×	Serum + RBC	LIM	Polybrene	Sodium citrate
**Volume (μL)**	10 + 5	100	×	100 + 50	600	100	100
**Time**	2 min		3–6 min	0.5 min	1 min	0.5 min (Centrifuge)	1 min
**Total time**	5–8 min			3–5 min			

**Table 3:** Comparison of the agglutination time between different concentrations of irregular antibody Anti-D and RBCs SI, SII, and SIII for screening.

	Ab-D Dilution	4 Times (Figure S9)	16 Times (Figure S10)	64 Times (Figure S11)	128 Times (Figure S12)	256 Times (Figure S13)
Time for Agglutination						
SI		3 min	4 min	4 min	10 min	15 min
SII		3 min	4 min	4 min	10 min	15 min
SIII		3 min	4 min	4 min	10 min	15 min

## Data Availability

Not applicable.

## Supplementary Materials

The following supporting information can be downloaded at: https://www.mdpi.com/article/10.3390/bios13090869/s1, Figure S1: The reaction of the irregular antibody Anti-D with the screening RBCs SI, SII, and SIII in the microdroplet with time; Figure S2: The reaction between irregular antibody Anti-C and screening RBCs in the microdroplet; Figure S3: The reaction of the irregular antibody Anti-E with the screening RBCs in the microdroplet; Figure S4: The reaction between the irregular antibody Anti-c and the screening RBCs in the microdroplet; Figure S5: The reaction between the irregular antibody Anti-e and the screening RBCs in the microdroplet; Figure S6: The reaction between the irregular antibody Anti-Le^a^ and the screening RBCs in the microdroplet; Figure S7: The reaction between the irregular antibody Anti-S and the screening RBCs in the microdroplet; Figure S8: The reaction between the irregular antibody Anti-M and the screening RBCs in the microdroplet; Figure S9: The reaction between the irregular antibody Anti-D that is diluted 4 times and the screening RBCs; Figure S10: The reaction between the irregular antibody Anti-D that is diluted 16 times and the screening RBCs; Figure S11: The reaction between irregular antibody Anti-D that is diluted 64 times and screening RBCs; Figure S12: The reactions between the irregular antibody Anti-D that is diluted 128 times and the screening RBCs; Figure S13: The reaction between the irregular antibody Anti-D that is diluted 256 times and the screening RBCs; Figure S14: The chip design of the accelerated RBC agglutination reaction; Figure S15: The agglutination reactions between RBCs and irregular Anti-M in the accelerated structured chip.
