# Supplementary material for: Irregular Antibody Screening Using a Microdroplet Platform
Source: Biosensors (Basel). 2023 Sep 4;13(9):869. doi: 10.3390/bios13090869 (PMC10526156; doi:10.3390/bios13090869)
Supplement: Supplementary file 1 [file biosensors-13-00869-s001.zip › biosensors-2531774-supplementary.pdf]

biosensors

MDPI

Supplementary Materials

Irregular antibody screening using a microdroplet platform

Ding-Ping Chen[1,2], Pei-Yu Wu[3] and Yen-Heng Lin[1,4,*]

1 Department of Laboratory Medicine, Chang Gung Memorial Hospital, Taoyuan City, Taiwan

2 Department of Medical Biotechnology and Laboratory Science, College of Medicine, Chang Gung University, Taoyuan City, Taiwan

3 Department of Electronic Engineering, Chang Gung University, Taoyuan City, Taiwan

4 Department of Biomedical Engineering, Chang Gung University, Taoyuan City, Taiwan

* Correspondence: yenheng@mail.cgu.edu.tw

Figure. S1

(a)

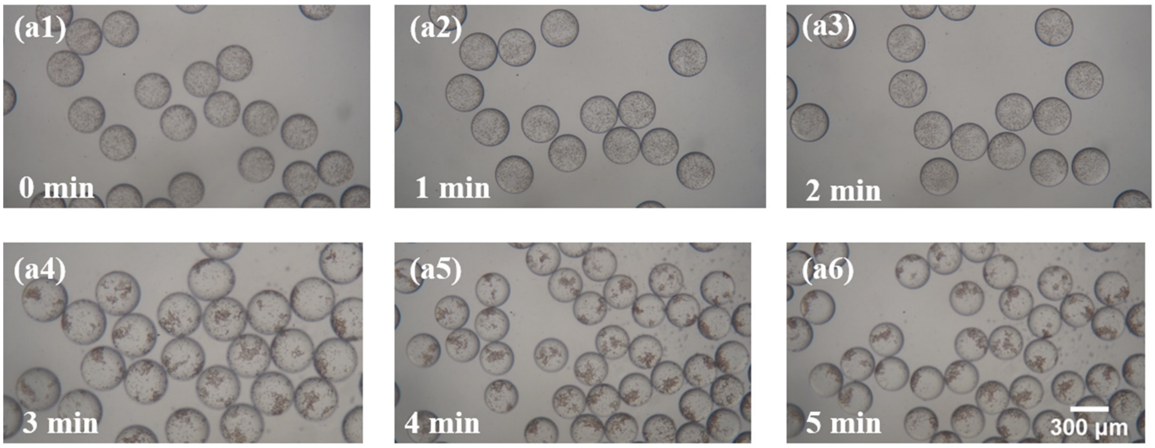

(b)

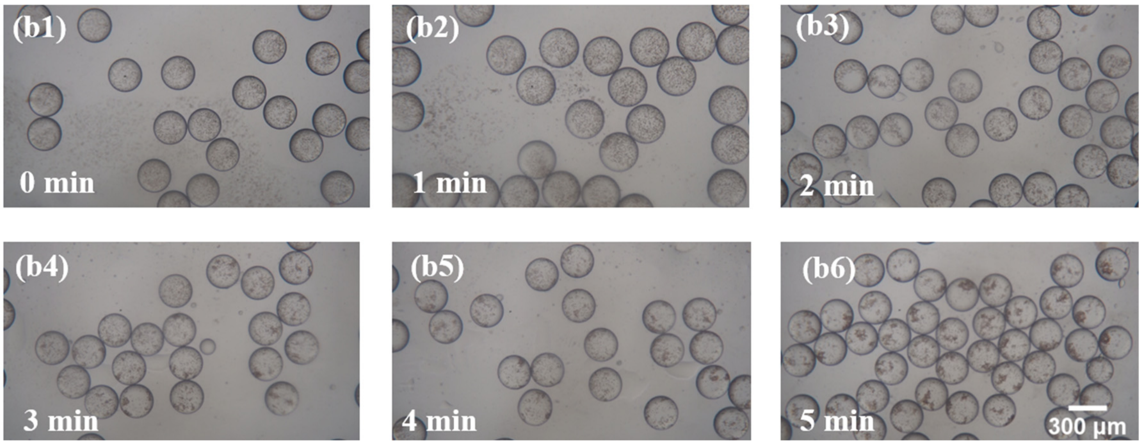

(c)

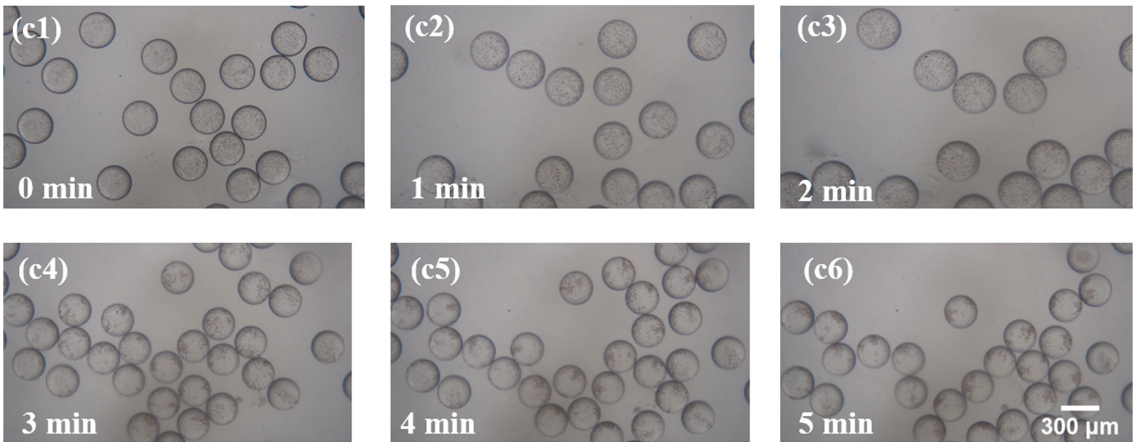

Figure S1 shows the reaction of the irregular antibody Anti-D with the screening RBCs SI, SII, and SIII in the microdroplet with time. (a) shows the reaction between SI and Anti-D in the microdroplet where a steady-state agglutination reaction can be observed at 3 min. (b) shows the reaction between SII and Anti-D, similar to the SI case above, where the agglutination reaction reaches a steady-state at 3 min. (c) shows the reaction between SIII and Anti-D, which has a reaction time similar to the previous two cases.

Figure. S2

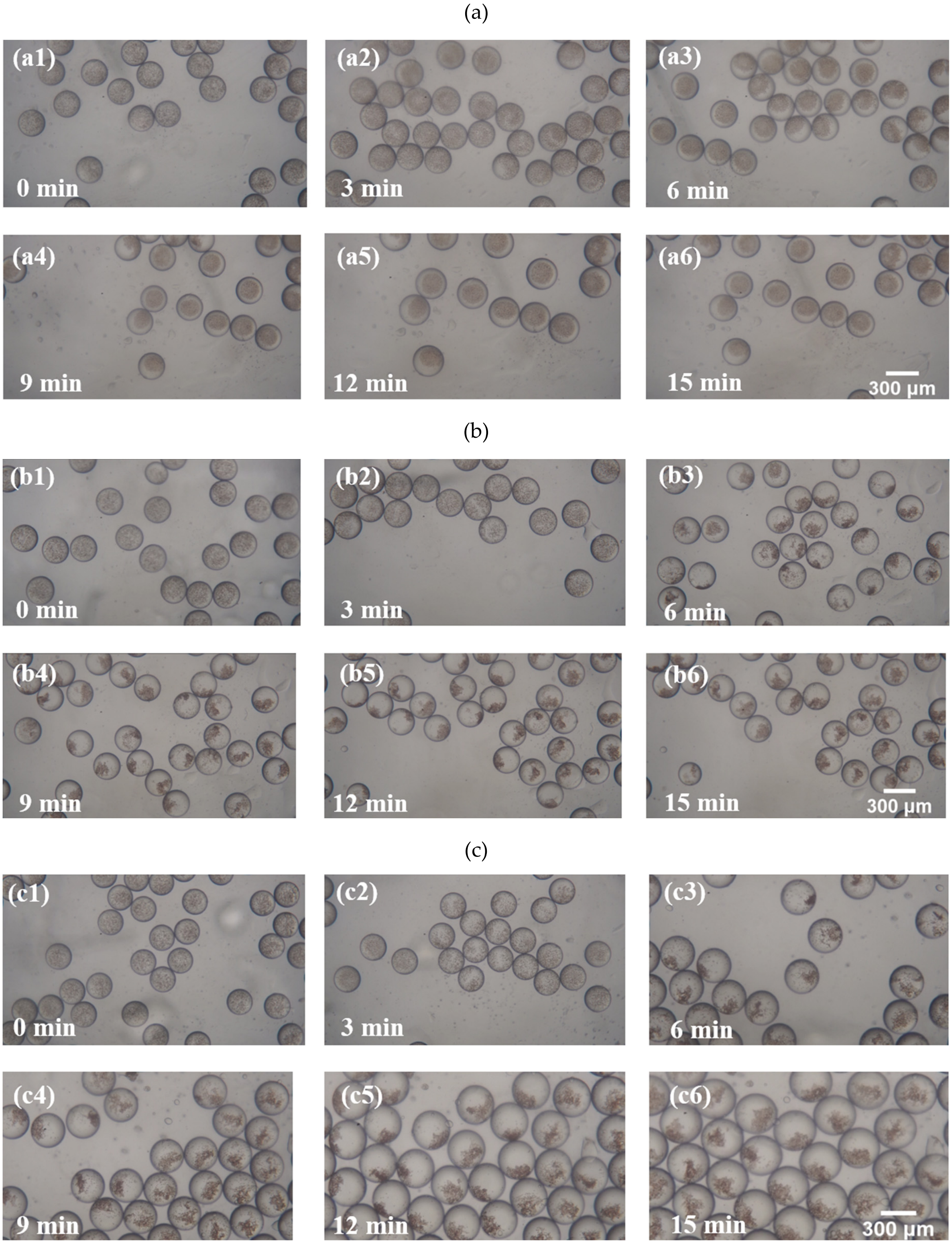

Figure S2 shows the reaction between irregular antibody Anti-C and screening RBCs SI, SII, and SIII in the microdroplet with time. (a) shows the reaction between SI and Anti-C in the microdroplet. Even after 15 min of reaction, the RBCs are still dispersed in the microdroplet without forming agglutination. (b) shows the reaction between SII and Anti-C, which reaches a steady-state agglutination reaction at 6 min. (c) shows the reaction between SIII and Anti-C, which similarly reaches a steady-state agglutination reaction at 6 min.

Figure. S3

(a)

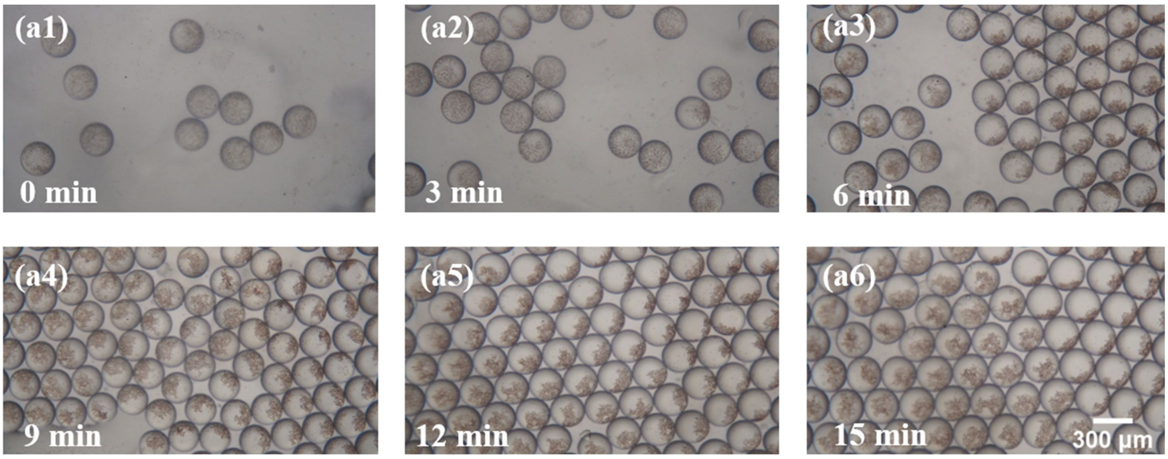

(b)

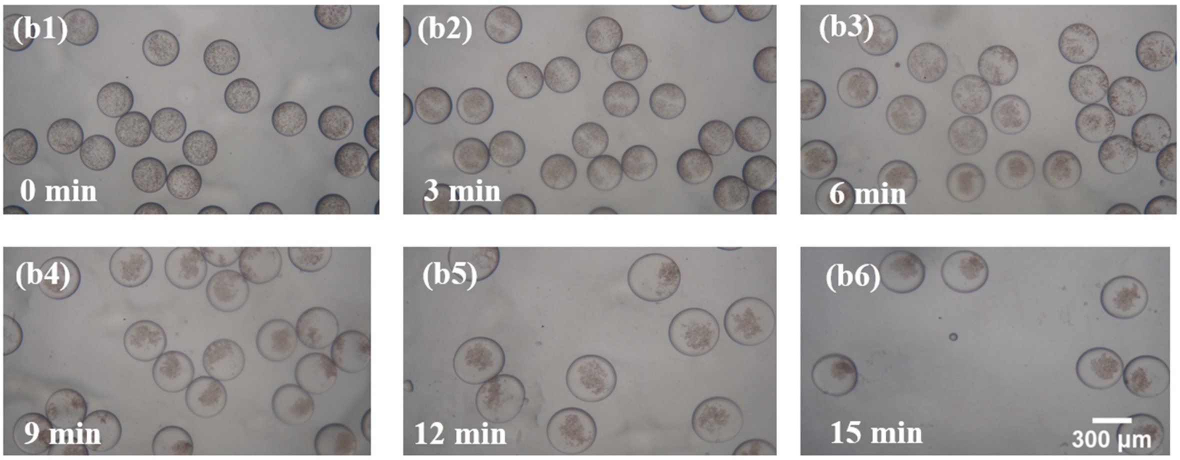

(c)

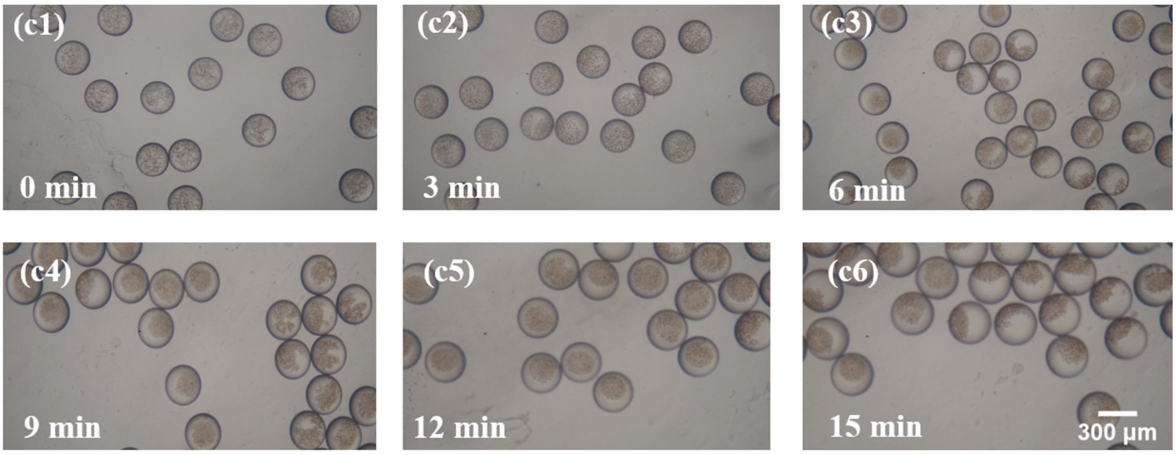

Figure S3 shows the reaction of the irregular antibody Anti-E with the screening RBCs SI, SII, and SIII in the microdroplet with time. (a) shows the reaction between SI and Anti-E in the microdroplet, where a steady-state agglutination reaction is reached at 6 min. (b)(c) show the reaction between SII, SIII, and Anti-E respectively, where it can be observed that even after 15 min of reaction, the RBCs are still dispersed in the microdroplets without forming agglutination.

Figure. S4

(a)

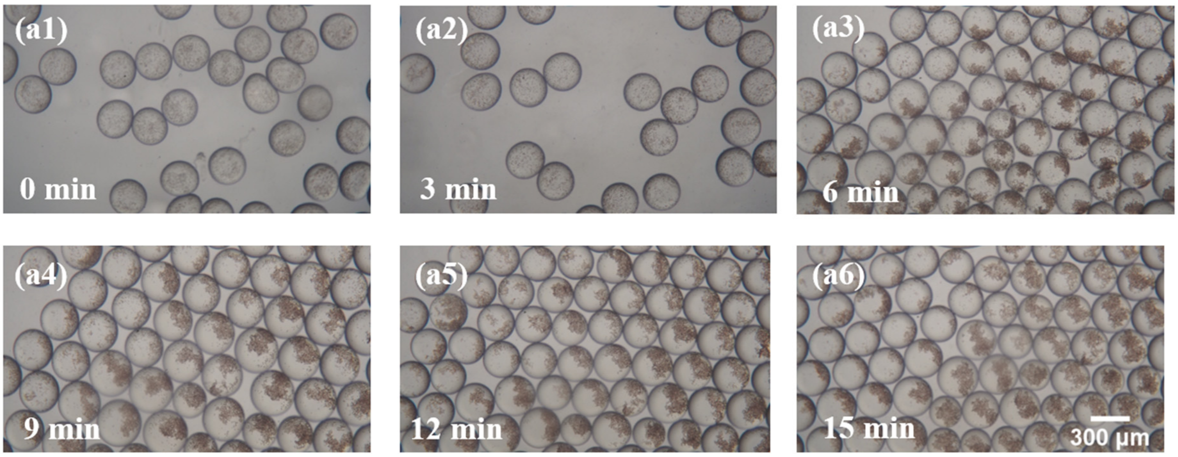

(b)

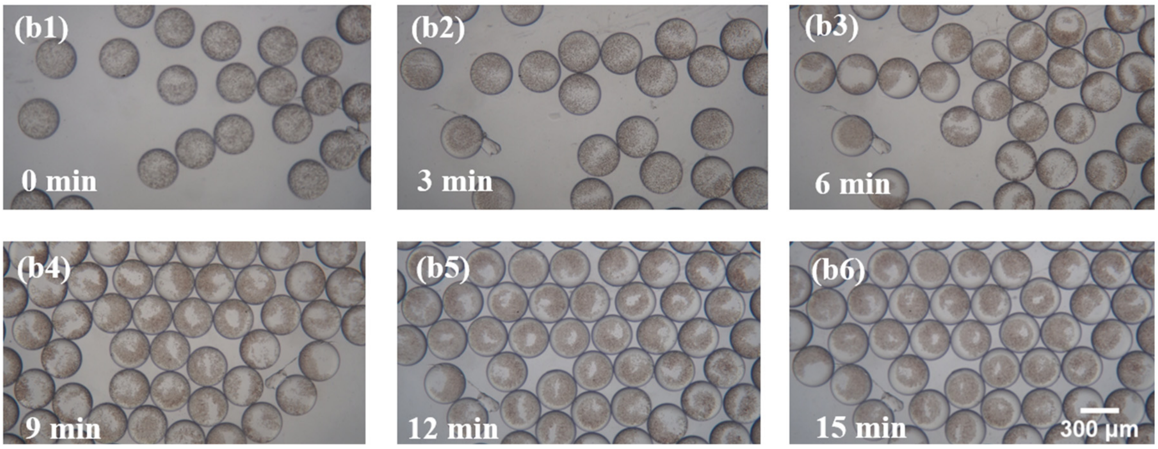

(c)

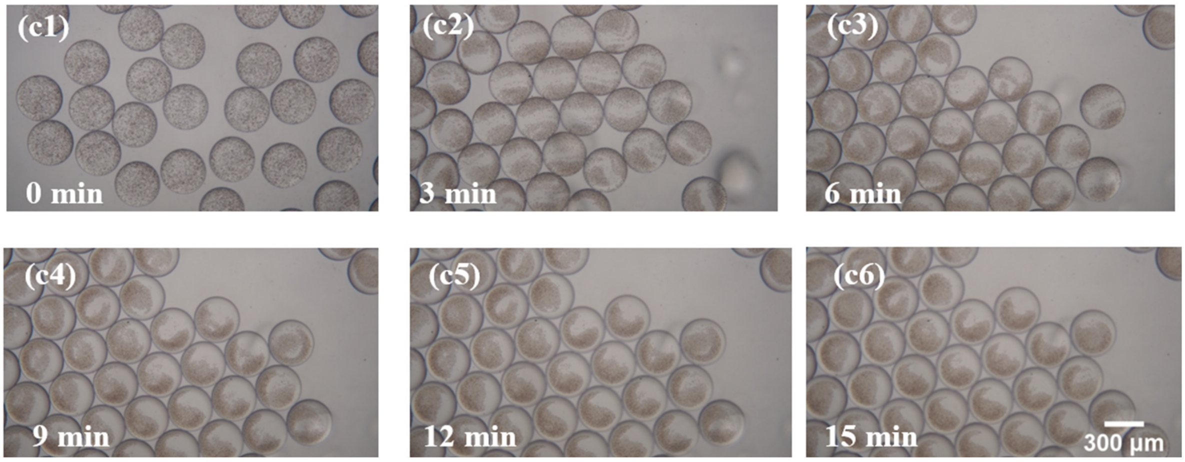

Figure S4 shows the reaction between the irregular antibody Anti-c and the screening RBCs SI, SII, and SIII in the microdroplet with time. (a) shows the reaction between SI and Anti-c in the microdroplet, where a steady-state agglutination reaction is reached at 6 min. (b)(c) shows the reaction between SII, SIII, and Anti-c respectively. Even after 15 min of reaction, the RBCs are still dispersed in the microdroplets without forming agglutination.

Figure. S5

(a)

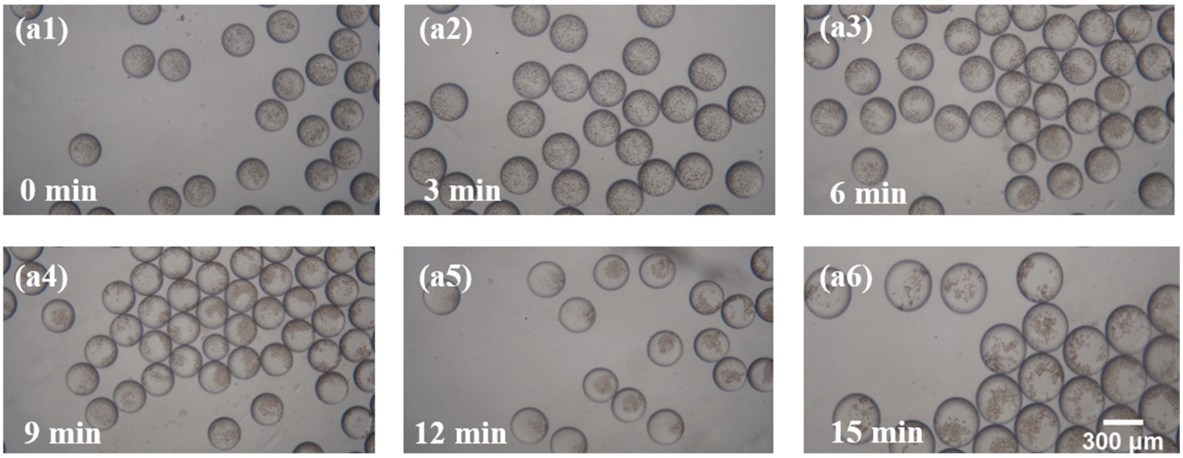

(b)

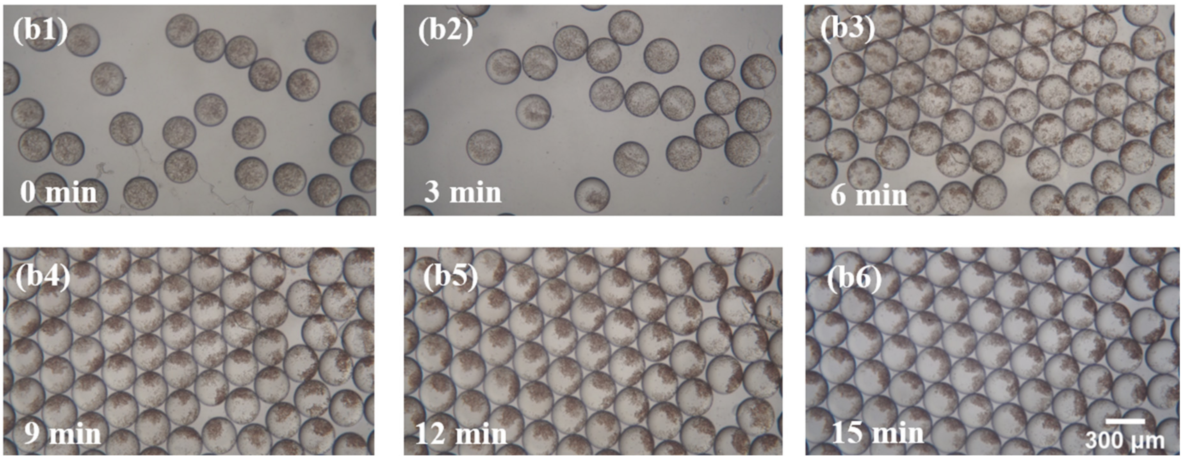

(c)

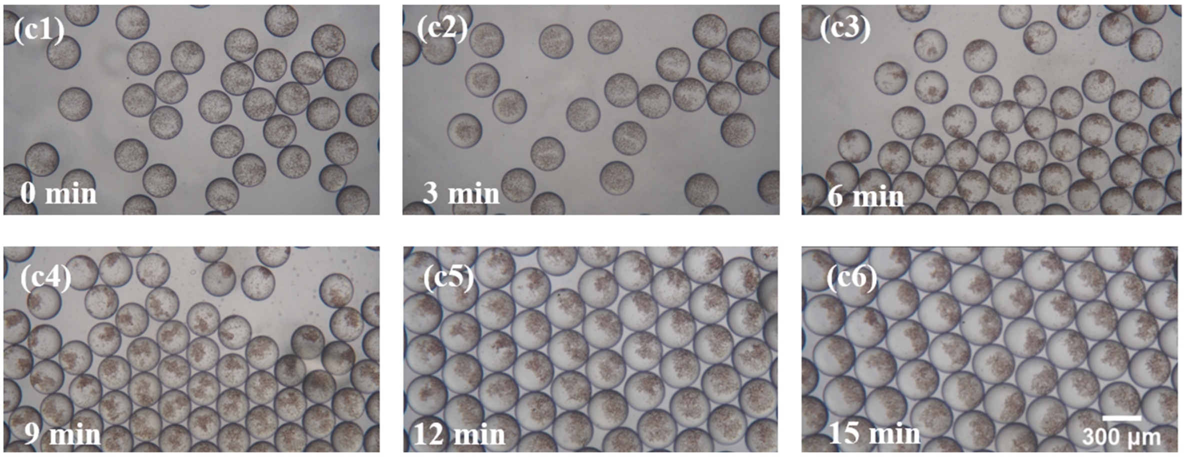

Figure S5 shows the reaction between the irregular antibody Anti-e and the RBCs SI, SII, and SIII for screening in the microdroplet with time. (a) shows the reaction between SI and Anti-e in the microdroplet. It can be observed that even after 15 min of reaction, the RBCs are still dispersed in the microdroplet without forming agglutination. (b) shows the reaction between SII and Anti-e, where a steady-state agglutination reaction is reached at 6 min. (c) shows the reaction between SIII and Anti-e, which is similar to that of SII.

Figure. S6

(a)

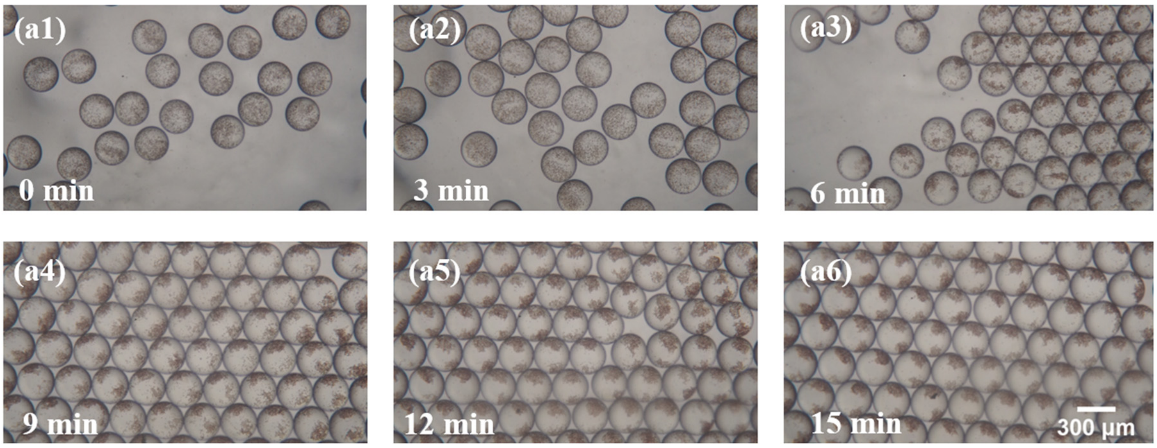

(b)

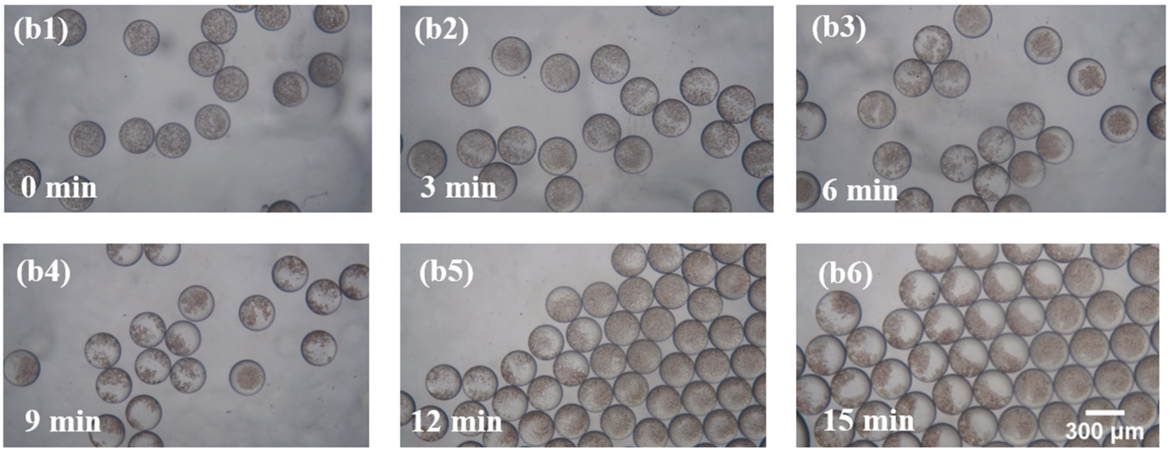

(c)

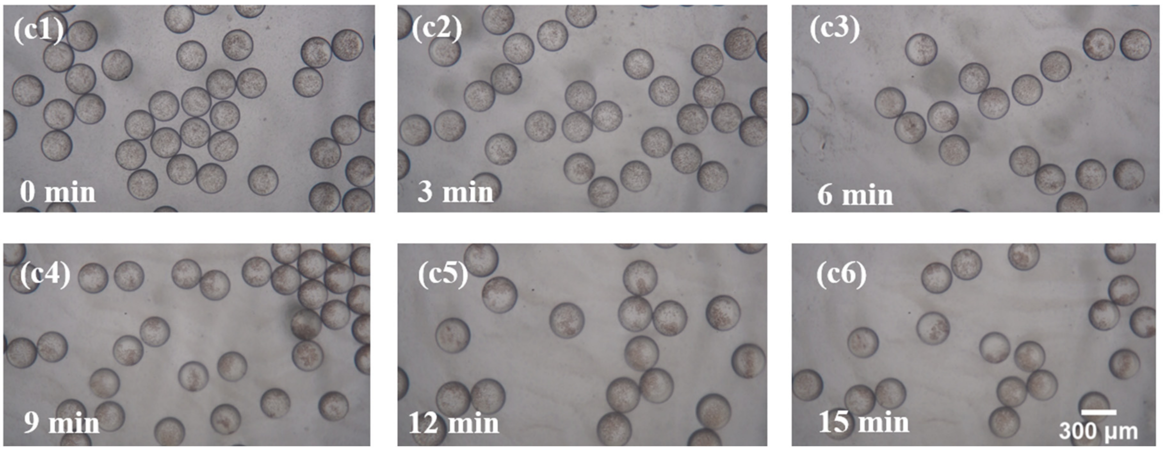

Figure S6 shows the reaction between the irregular antibody Anti-Le^a and the RBCs SI, SII, and SIII for screening in the microdroplet with time. (a) shows the reaction between SI and Anti-Le^a in the microdroplet, where a steady-state agglutination reaction is reached at approximately 6 min. (b)(c) show the reaction between SII, SIII, and Anti-Le^a, respectively. It can be observed that even after 15 min of reaction, the RBCs are still dispersed in the microdroplets without forming agglutination.

Figure. S7

(a)

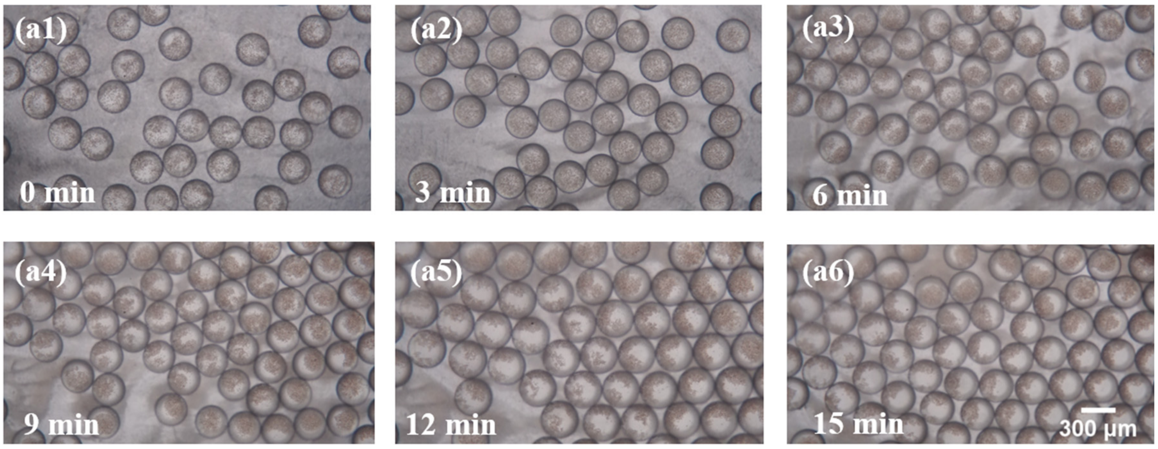

(b)

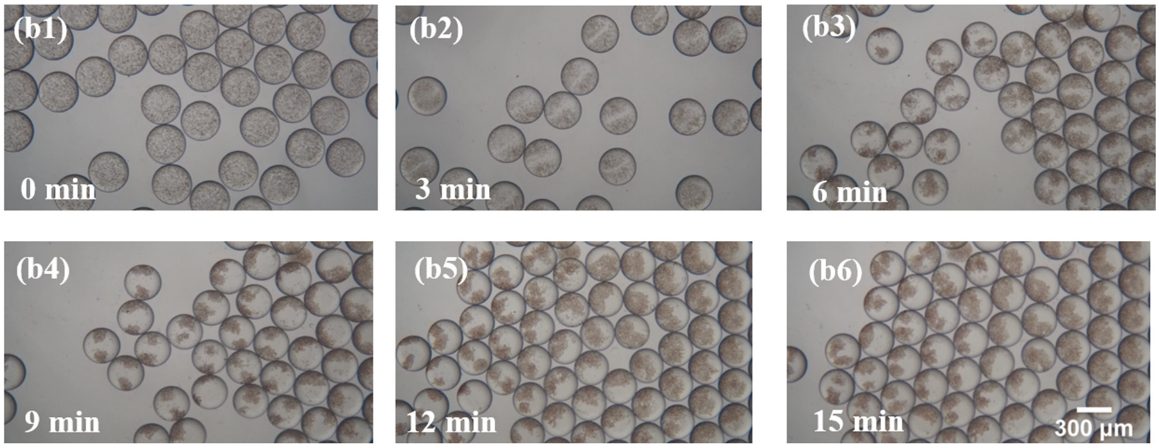

(c)

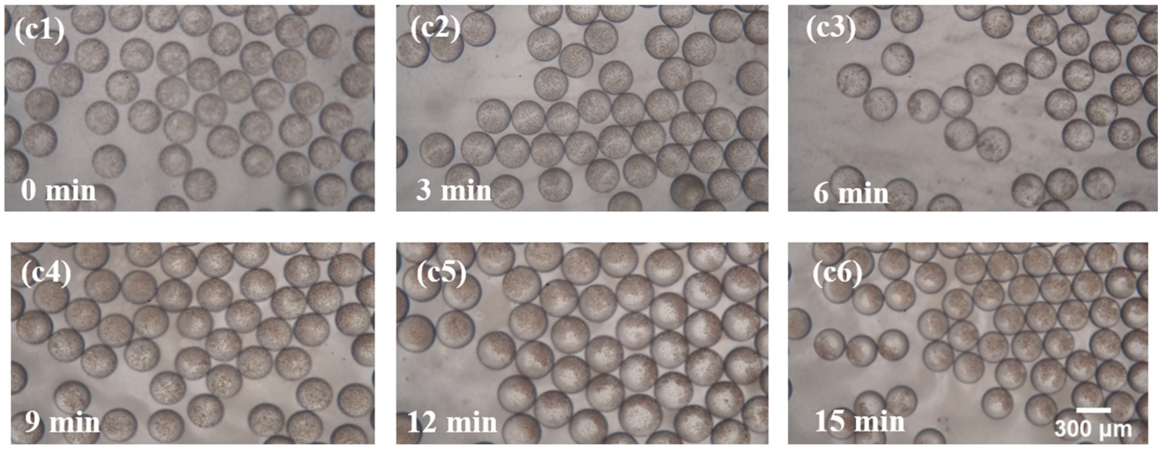

Figure S7 shows the reaction between the irregular antibody Anti-S and the screening RBCs SI, SII, and SIII. (a) shows the reaction between SI and Anti-S in the microdroplet. It can be observed that even after 15 min of reaction, the RBCs are still dispersed in the microdroplet without forming agglutination. (b) shows the reaction between SII and Anti-S, where a steady-state agglutination reaction is reached at 6 min. (c) shows the reaction between SIII and Anti-S. It can be observed that even after 15 min of reaction, the RBCs are still dispersed without forming agglutination.

Figure. S8

(a)

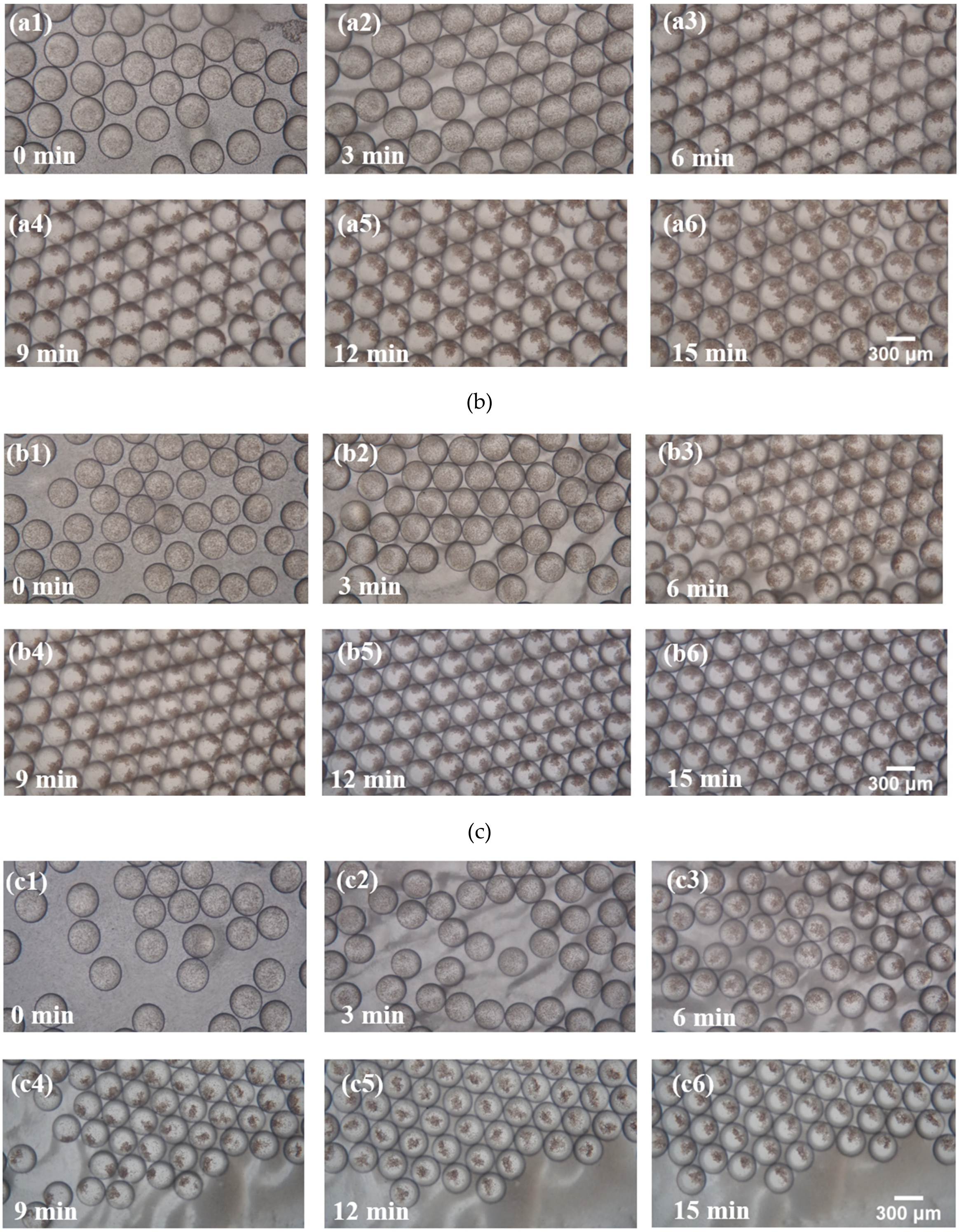

Figure. S8 (a) (b) (c) show the reaction between SI, SII, SIII, and Anti-M in the microdroplet, respectively, where all three reactions reach a steady-state agglutination reaction at approximately 6 min.

Figure. S9

(a)

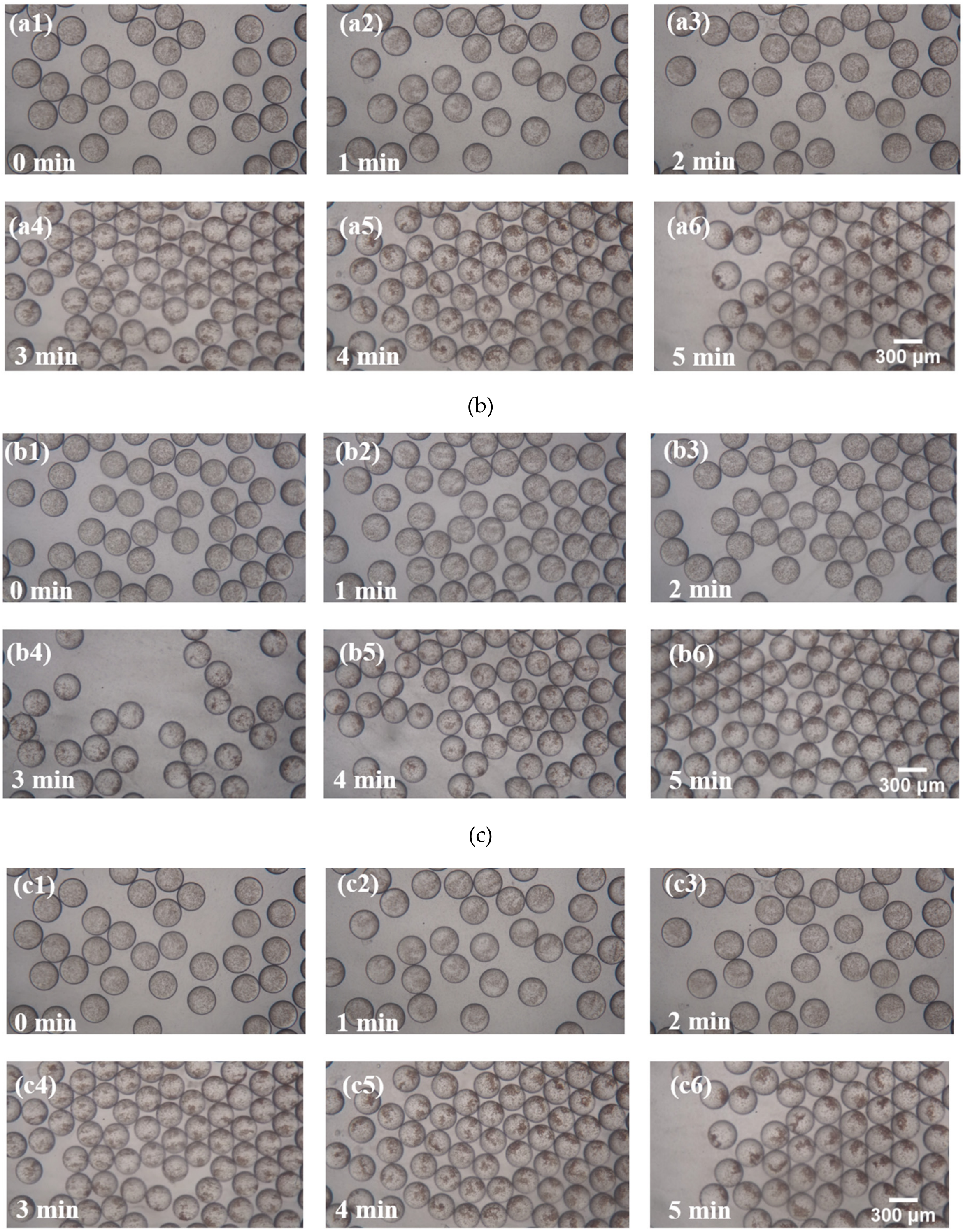

Figure S9 shows the reaction between the irregular antibody Anti-D that is diluted 4 times and the screening RBCs (a) SI (b) SII (c) SIII, respectively. It can be seen that the agglutination reaction of these three combinations reaches a steady-state agglutination at approximately 3 min.

Figure. S10

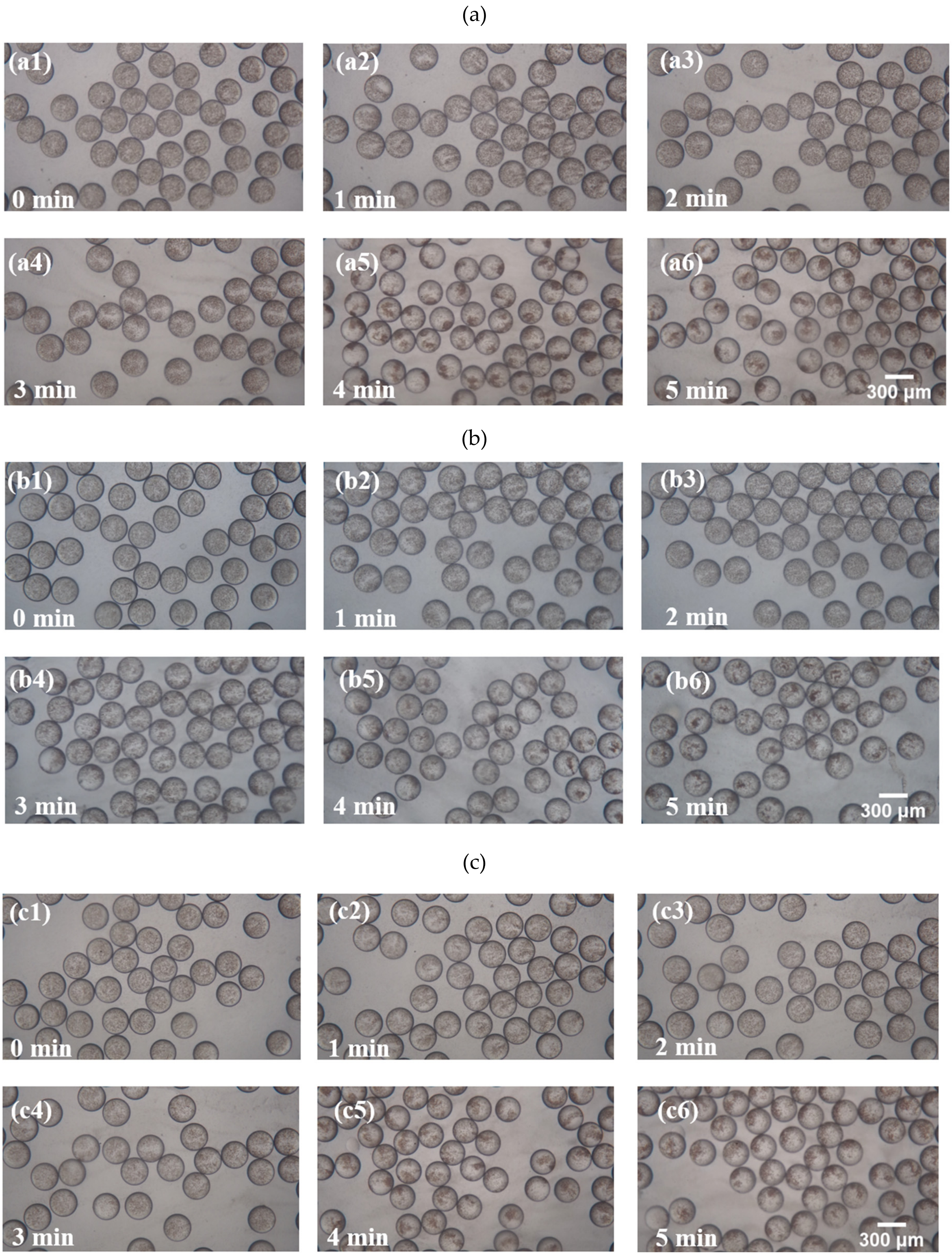

Figure S10 shows the reaction between the irregular antibodies Anti-D that is diluted 16 times with the screening RBCs (a) SI (b) SII (c) SIII, respectively. It can be seen that the agglutination reaction of these three combinations reaches a steady-state agglutination at approximately 4 min.

Figure. S11

(a)

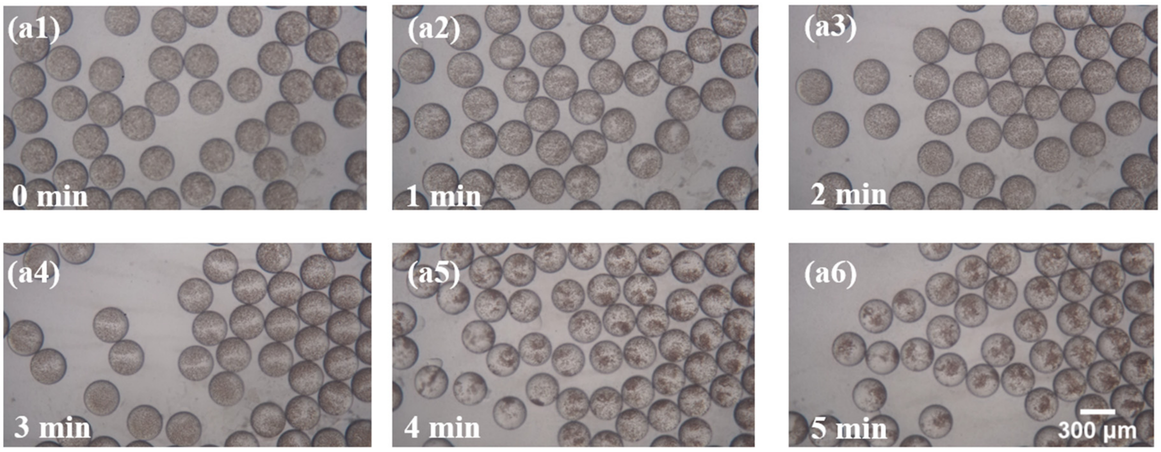

(b)

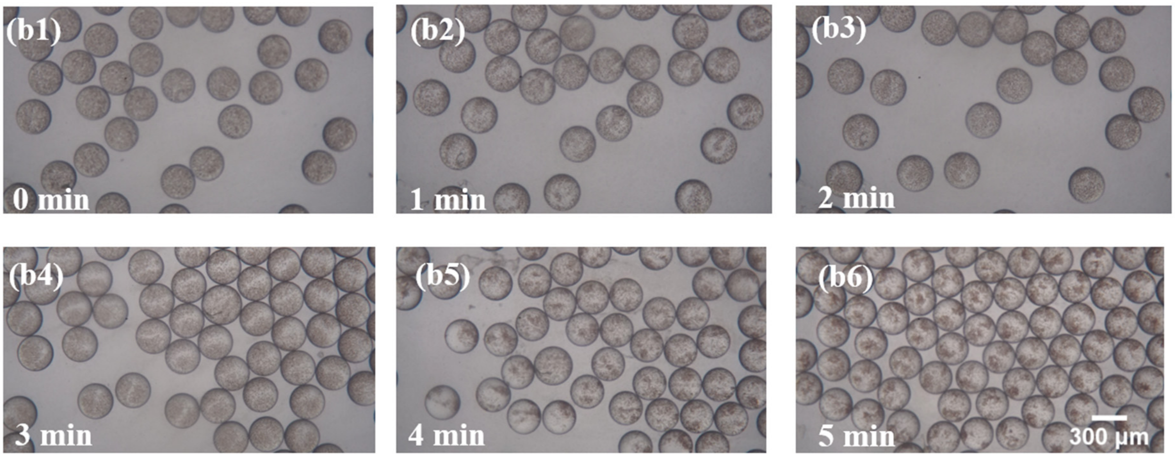

(c)

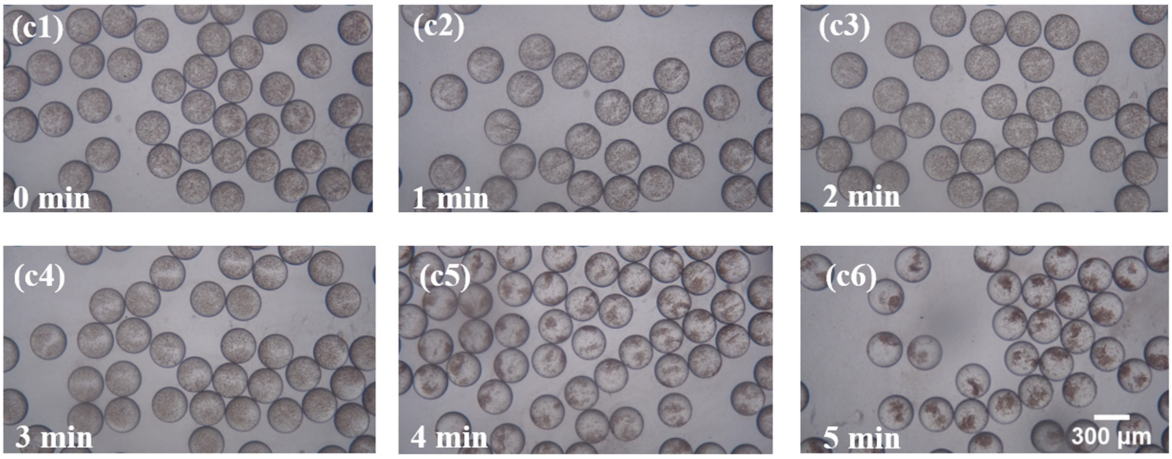

Figure S11 shows the reaction between irregular antibodies Anti-D that is diluted 64 times and screening RBCs (a) SI (b) SII (c) SIII, respectively. It was observed that the agglutination reaction of these three combinations reached steady-state agglutination at approximately 4 min.

Figure. S12

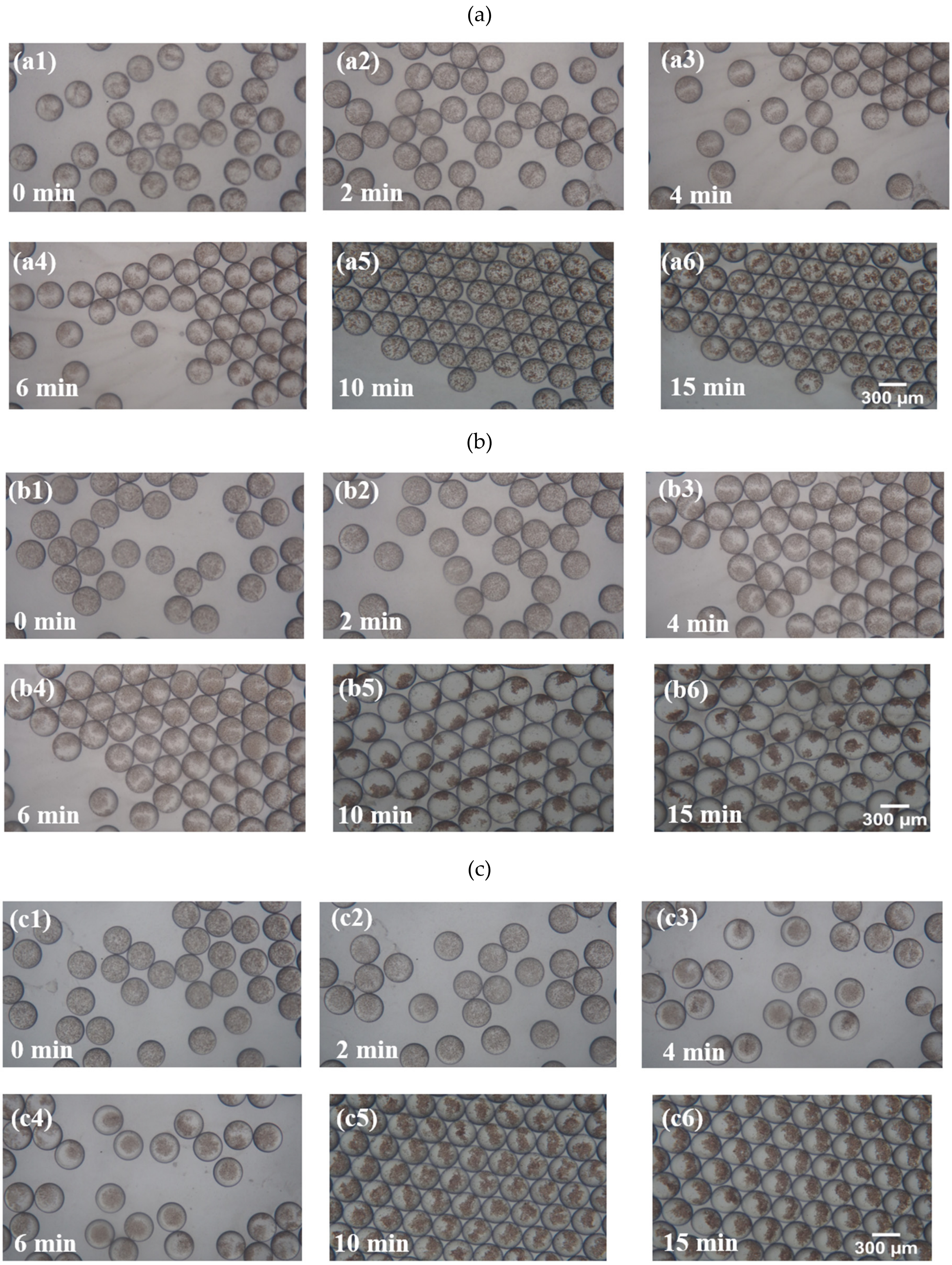

Figure S12 shows the reactions between the irregular antibodies Anti-D that is diluted 128 times and the screening RBCs (a) SI (b) SII (c) SIII, respectively. It can be seen that the agglutination reaction of these three combinations reaches a steady-state agglutination at approximately 10 min.

Figure. S13

(a)

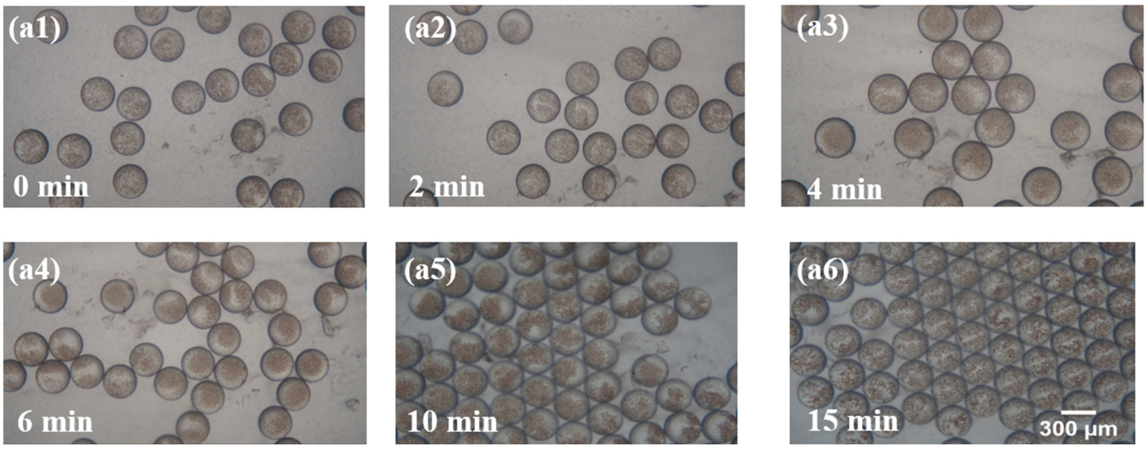

(b)

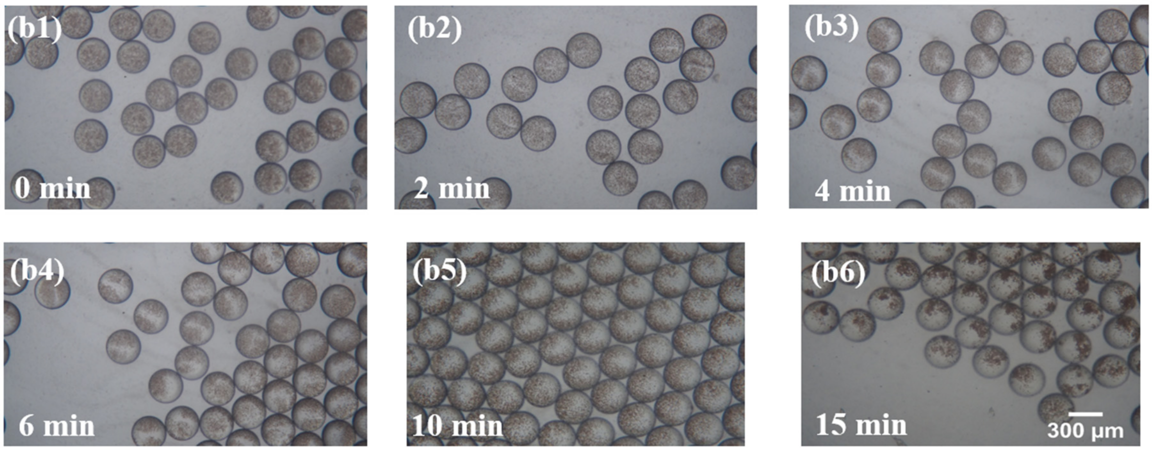

(c)

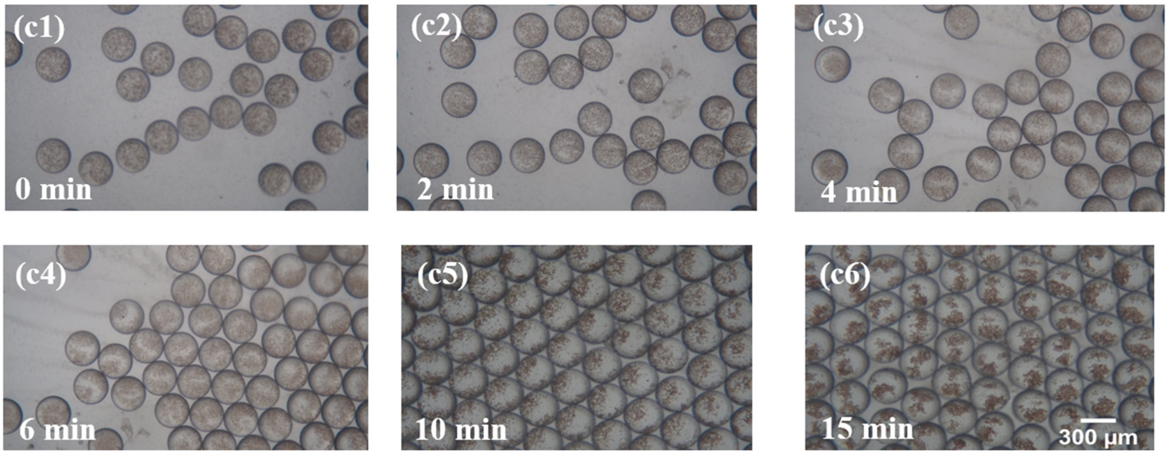

Figure S13 shows the reaction between the irregular antibodies Anti-D that is diluted 256 times and the screening RBCs (a) SI (b) SII (c) SIII, respectively. It can be seen that the agglutination reaction of these three combinations reaches a steady-state agglutination at approximately 15 min.

Figure. S14

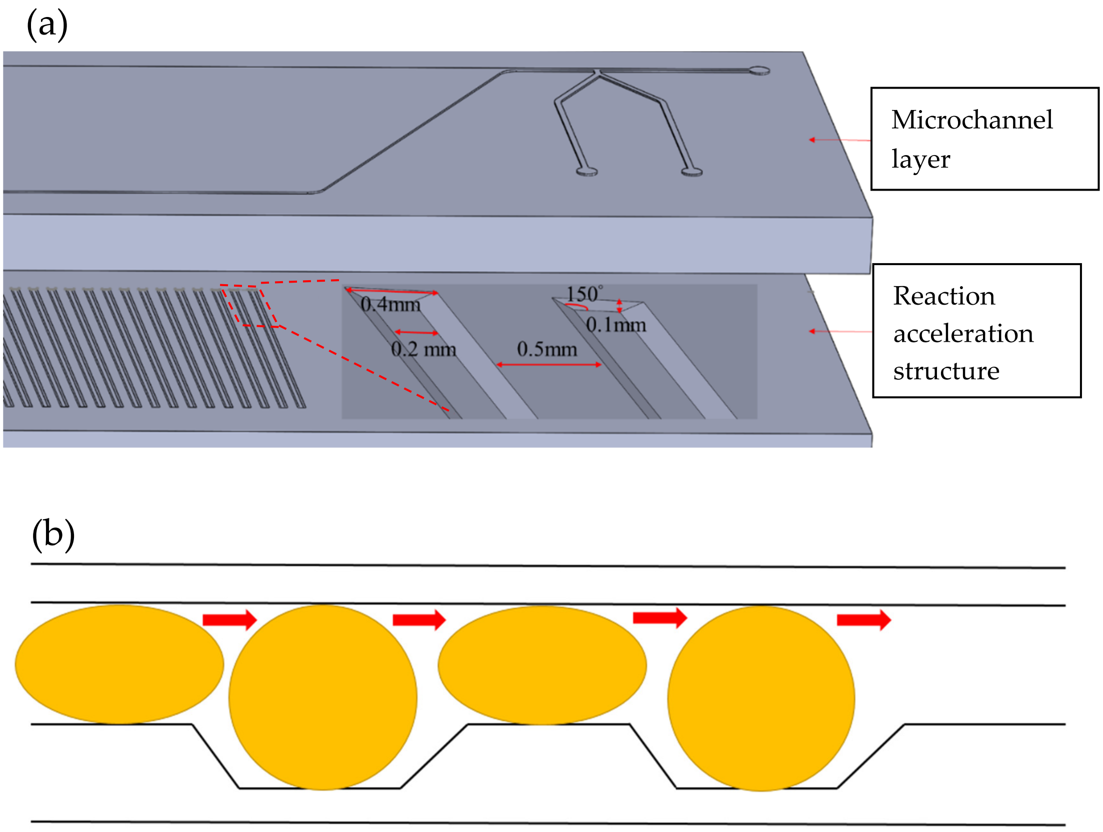

Figure. S14 (a) The chip design of the accelerated RBC agglutination reaction. We employed the original flow channel design, and a periodic groove structure was added to the lower plate of the reaction zone to create a periodic change in the height of the flow channel. (b) Schematic diagram of the reaction acceleration principle. The microdroplets flow through the high and low flow channels sequentially, causing changes in microdroplet shape and squeezing the internal fluid of the microdroplets to accelerate the RBC agglutination reaction.

Figure. S15

(a)

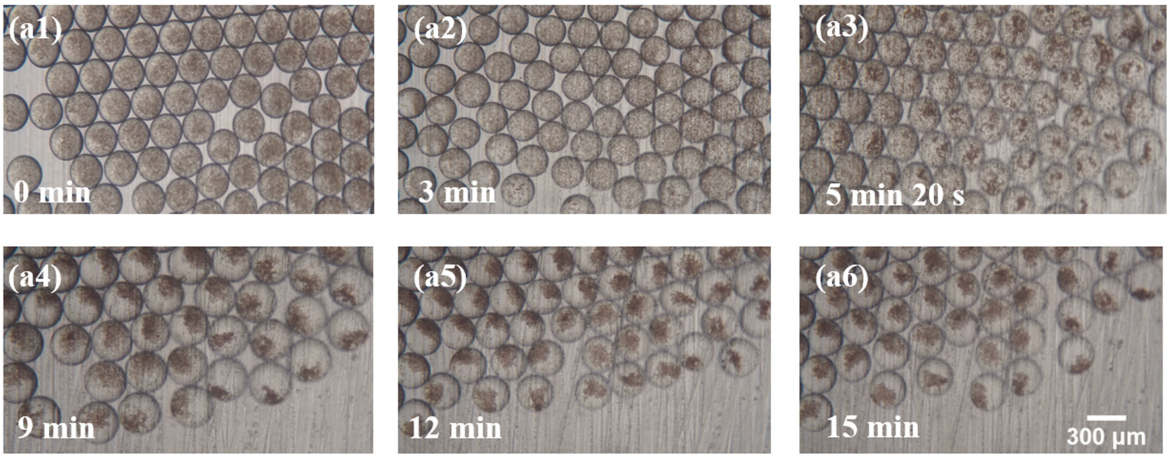

(b)

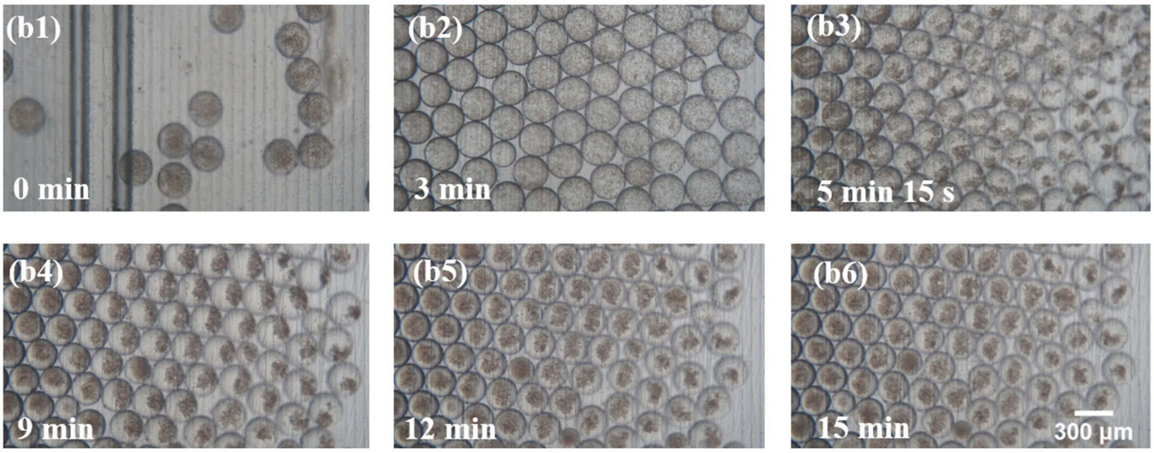

(c)

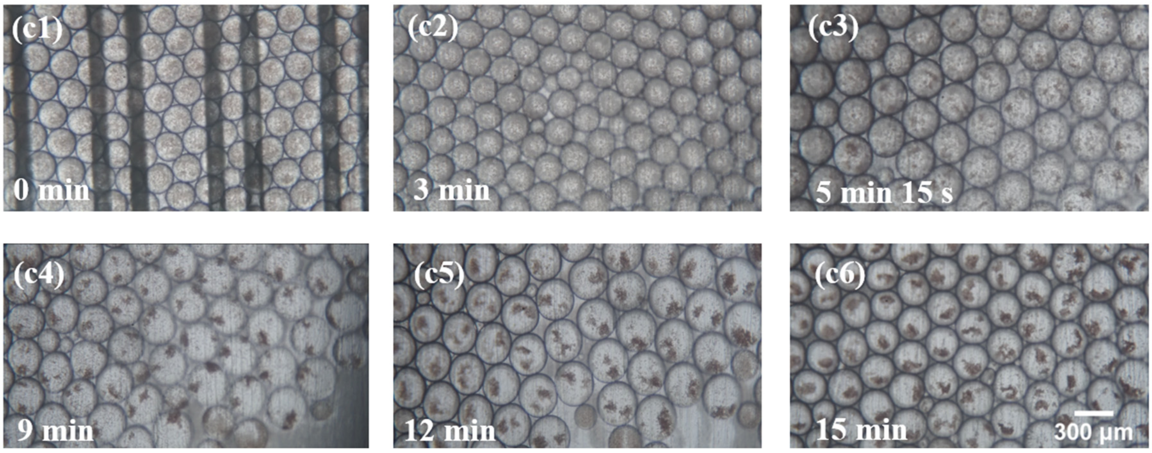

Figure S15 (a) (b) (c) show the RBC agglutination reactions between RBCs SI, SII, SIII, and irregular Anti-M in the accelerated structured chip. It can be seen that all three reactions have reached steady-state agglutination in approximately 5 min, which is approximately 16.7% faster than the reaction rate of the chip without the accelerated structure.
